# KDM5-mediated activation of genes required for mitochondrial biology is necessary for viability in *Drosophila*

**DOI:** 10.1242/dev.202024

**Published:** 2023-11-06

**Authors:** Michael F. Rogers, Owen J. Marshall, Julie Secombe

**Affiliations:** ^1^ Department of Genetics, Albert Einstein College of Medicine, Bronx, NY 10461, USA; ^2^ Menzies Institute for Medical Research, University of Tasmania, Hobart TAS 7000, Australia; ^3^ Dominick P. Purpura Department of Neuroscience, Albert Einstein College of Medicine, Bronx, NY 10461, USA

**Keywords:** KDM5, Demethylase, Transcription, Prothoracic gland, Mitochondria

## Abstract

Histone-modifying proteins play important roles in the precise regulation of the transcriptional programs that coordinate development. KDM5 family proteins interact with chromatin through demethylation of H3K4me3 as well as demethylase-independent mechanisms that remain less understood. To gain fundamental insights into the transcriptional activities of KDM5 proteins, we examined the essential roles of the single *Drosophila Kdm5* ortholog during development. KDM5 performs crucial functions in the larval neuroendocrine prothoracic gland, providing a model to study its role in regulating key gene expression programs. Integrating genome binding and transcriptomic data, we identify that KDM5 regulates the expression of genes required for the function and maintenance of mitochondria, and we find that loss of KDM5 causes morphological changes to mitochondria. This is key to the developmental functions of KDM5, as expression of the mitochondrial biogenesis transcription factor Ets97D, homolog of GABPα, is able to suppress the altered mitochondrial morphology as well as the lethality of *Kdm5* null animals. Together, these data establish KDM5-mediated cellular functions that are important for normal development and could contribute to KDM5-linked disorders when dysregulated.

## Teaser

**Summary:** Transcriptional and cellular studies show that KDM5-mediated regulation of genes necessary for the function of mitochondria is essential for animal development.

## INTRODUCTION

Transcriptional regulators function as powerful gatekeepers that enable cells to access and utilize the information stored in the genome. The dynamics of chromatin organization and transcriptional mechanisms must therefore be carefully coordinated to orchestrate the gene expression programs required for proper development. Conversely, improper function of transcriptional regulators can underlie the defective cellular processes that lead to dysfunction and disease (Lee and Young, 2013; Mirabella et al., 2016). Within this realm of biology, chromatin-modifying proteins interface with histone protein tails through writing, reading and erasing post-translational modifications to organize gene expression. Lysine demethylase 5 (KDM5) proteins are one such family of chromatin-modifiers that are named for their ability to remove trimethylation of lysine 4 on histone H3 (H3K4me3), a mark generally found near the transcriptional start sites of actively expressed genes (Chan et al., 2022).

Mammalian cells encode four paralogous KDM5 proteins: KDM5A, KDM5B, KDM5C and KDM5D. The importance of gene regulation by KDM5 family proteins is demonstrated by their links to human disorders. Altered expression of each of the four KDM5 genes has been observed across a variety of cancer types, of which breast and prostate cancer are the most well characterized (Ohguchi and Ohguchi, 2022; Blair et al., 2011). The relationship between KDM5A, KDM5B and tumorigenesis appears to be primarily oncogenic, with a range of cancers showing increased expression of either of these two paralogs. Rather than being linked to the regulation of a single process, KDM5A and KDM5B contribute to many facets of tumorigenesis, including the regulation of genes linked to cell cycle control, DNA repair and angiogenesis (Ohguchi et al., 2021; Taylor-Papadimitriou and Burchell, 2022; Ohguchi and Ohguchi, 2022; Yoo et al., 2022). The roles of KDM5C and KDM5D in malignancies are less defined, although, in contrast to KDM5A and KDM5B, it is generally reduction of these proteins that is observed in cancers, most notably renal carcinomas (Tricarico et al., 2020). The genetic association between KDM5 proteins and neurodevelopmental disorders, including intellectual disability and autism spectrum disorders, is more clearly caused by loss-of-function variants in *KDM5A*, *KDM5B* or *KDM5C* (Hatch and Secombe, 2021; Yoo et al., 2022). Consistent with this, mouse and cell culture models have shown that Kdm5a, Kdm5b and/or Kdm5c are needed for proper neuronal differentiation and morphology (El Hayek et al., 2020; Harrington et al., 2022; Iwase et al., 2017, 2016). However, although KDM5 proteins are clearly required for normal brain function, the transcriptional programs required for typical cognitive development remain unknown. It also remains unclear whether similar or distinct transcriptional programs etiologically link KDM5 to malignancies and to brain development. In this regard, it is notable that, although cancer and intellectual disability have vastly different clinical manifestations, alterations in the activity of other regulatory factors, such as members of the MAPK and PI3K signaling cascades, are also linked to these same two groups of disorders (Borrie et al., 2017). Thus, it remains possible that dysregulation of overlapping pathways contributes to both tumorigenesis and altered cognition.

Defining how changes to KDM5 protein function leads to cancer or intellectual disability would be greatly facilitated by efforts to understand their fundamental transcriptional activities. To date, attempts to define these links have focused on their canonical histone demethylase activity. However, KDM5 and other chromatin-modifying proteins also perform important non-catalytic gene regulatory functions that play roles in both cancer and intellectual disability (Iwase et al., 2007; Cao et al., 2014; Vallianatos et al., 2018; Aubert et al., 2019; Ohguchi and Ohguchi, 2022; Morgan and Shilatifard, 2023). This is also true in *Drosophila melanogaster*, in which one gene encodes a single KDM5 protein that is likely to function by incorporating activities of all four mammalian paralogs. A null allele in *Drosophila Kdm5* (*Kdm5^140^*) causes lethality during development (Drelon et al., 2018), highlighting its crucial role in developmental processes. The essential functions of KDM5 are independent of its enzymatic demethylase function, however, as animals harboring loss-of-function mutations in the enzymatic Jumonji C (JmjC) domain survive to adulthood (Li et al., 2010; Drelon et al., 2018). Characterizing the role of KDM5 during *Drosophila* development therefore provides an opportunity to uncover new pathways and gene-regulatory mechanisms that will expand our understanding of this family of multi-domain proteins.

Several cell types in *Drosophila* require KDM5 during development. Consistent with the established link between genetic variants in human *KDM5* genes and intellectual disability, KDM5 is necessary for proper neuronal development and functioning (Zamurrad et al., 2018; Belalcazar et al., 2021; Hatch et al., 2021). However, these neuronal activities of KDM5 are not necessarily involved in its essential developmental functions, as restoring *Kdm5* expression pan-neuronally does not rescue lethality (Drelon et al., 2019). KDM5 has also been linked to immune function in larval hemocytes, but, in a similar manner to neurons, this cell type does not account for its essential activities (Moran et al., 2015; Drelon et al., 2019). The only single tissue in which re-expression of *Kdm5* is sufficient to rescue lethality is the prothoracic gland (Drelon et al., 2019). *Kdm5^140^* animals rescued by prothoracic gland-specific *Kdm5* expression develop into adult flies; however, they survive at a lower frequency than animals expressing *Kdm5* ubiquitously, which indicates that KDM5 plays important functions in other tissues. Nevertheless, this demonstrates that, within prothoracic gland cells, KDM5 regulates the expression of genes crucial to proper organismal development.

A neuroendocrine tissue, the prothoracic gland serves as a master coordinator of numerous intracellular processes, tissue growth, and organismal transitions that are essential to development through its production of the steroid hormone ecdysone (Texada et al., 2020; Kamiyama and Niwa, 2022). This tissue is also a well-established model for understanding how key signaling pathways are integrated to govern hormone dynamics and animal maturation, including the MAPK, Salvador-Warts-Hippo-Yorkie (SHW), target of rapamycin (TOR) and insulin and insulin-like signaling (IIS) cascades. These pathways converge on processes such as autophagy and endoreplication that are important for regulating metabolism and hormone production in the prothoracic gland (Moeller et al., 2017; Texada et al., 2019, 2020; Zeng et al., 2020). Like KDM5 family proteins, the dysregulation of many of these pathways is implicated in human disorders, including cancer and neurodevelopmental disorders (Vithayathil et al., 2018; Kim and Choi, 2010; Williamson et al., 2014; Zanconato et al., 2016; Tian et al., 2019). We previously showed that KDM5 regulates larval growth rate through promotion of prothoracic gland cell endoreplication, which is thought to increase expression of ecdysone biosynthetic factors (Drelon et al., 2019; Ohhara et al., 2017, 2019). The prothoracic gland functions of KDM5 in facilitating larval growth, however, are separate from its role in survival, as restoring normal developmental timing to *Kdm5* mutant animals does not alter their lethality. The role of KDM5 in viability involves MAPK signaling, as *Kdm5* null mutant animals show decreased MAPK signaling and activating this pathway suppresses *Kdm5* mutant lethality. However, whether this effect is specific to the MAPK pathway, and which downstream cellular processes link KDM5, MAPK and viability, remain to be established.

Here, we examine KDM5 function in the prothoracic gland to understand broadly how this chromatin modifier regulates crucial cellular processes. Extending our previous studies, we explore the role of the MAPK and parallel pathways in mediating the lethality caused by loss of *Kdm5*. We additionally take unbiased approaches to define the transcriptional targets of KDM5. Among these targets, we identified mitochondrial biology as a candidate process for which KDM5-mediated regulation could play crucial roles during development. Reinforcing these connections, the lethality of the *Kdm5* null allele is suppressed by expression of Ets97D (also known as Delg), the *Drosophila* homolog of GA binding protein transcription factor subunit alpha (GABPα), a known activator of genes necessary for mitochondrial biosynthesis. Furthermore, prothoracic gland cells of *Kdm5* mutant animals show altered mitochondrial morphology dynamics that are suppressed by expression of Ets97D. Together, this study provides new insights into the link between KDM5-regulated transcription, mitochondrial function, and vital cellular processes needed for development.

## RESULTS

### Activation of MAPK signaling suppresses *Kdm5* null lethality independently of autophagy regulation

**Fig. 1.**
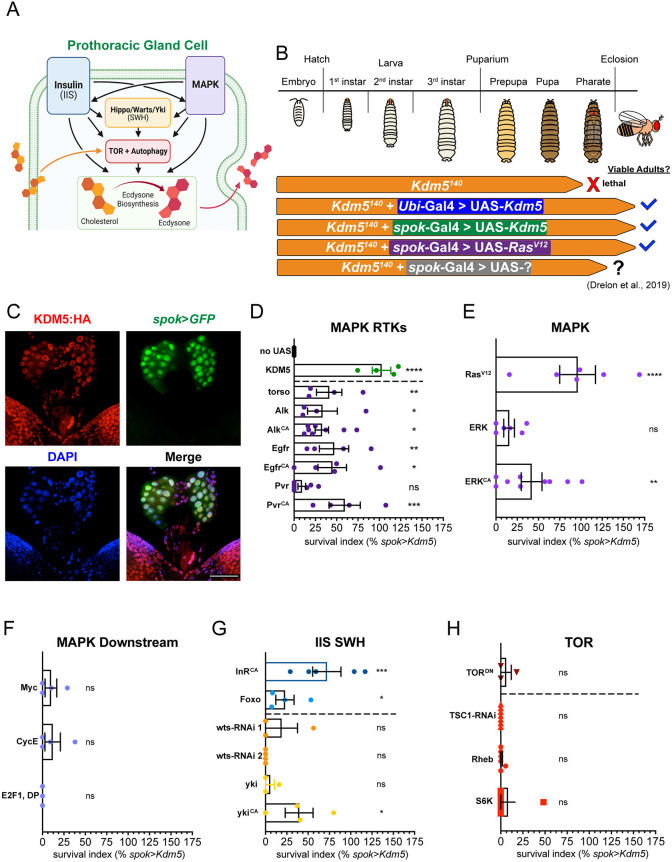
**MAPK signaling robustly suppresses *Kdm5^140^* lethality independently of autophagy regulation.** (A) Major signaling pathways regulating prothoracic gland cell function. Potential crosstalk is indicated by black arrows. (B) Summary of *Kdm5^140^* pupal pharate lethality suppression by transgene expression (from Drelon et al., 2019). (C) Maximum intensity *z*-projection of larval brain-ring gland complex. Anti-HA shows endogenously tagged KDM5:HA in nuclei (DAPI) of the prothoracic gland (*spok*>*GFP*). Scale bar: 50 μm. Images are representative of 9 samples. (D-H) Quantification of survival index for *spok*-Gal4-driven expression of transgenes in *Kdm5^140^* background relative to *spok*-Gal4>UAS-*Kdm5* (green data points in D). ****P<0.0001, ***P<0.001, **P<0.01, *P<0.05; ns, not significant [Fisher's exact test of each transgene compared with ‘no UAS’ control (black data points in D)]. Error bars represent s.e.m. (D) Quantification of survival index for expression of MAPK-activating RTKs. *n*=300, 380, 562, 575, 685, 681, 191, 722, 486, respectively. (E) Quantification of survival index for expression of MAPK signaling components. *n*=467, 613, 800, respectively. (F) Quantification of survival index for expression of candidate factors downstream of MAPK. *n*=484, 368, 283, respectively. (G) Quantification of survival index for expression of IIS and SWH signaling components. *n*=440, 921, 435, 346, 292, 360, respectively. (H) Quantification of survival index for expression of TOR signaling components. *n*=352, 406, 926, 868, respectively. Dashed lines separate transgene categories in a given plot, specifically negative and positive controls from MAPK RTKs (D), IIS from SWH components (G), and transgenes with repressive from those with activating effects on TOR signaling (H).

To understand better the crucial developmental roles of KDM5, we investigated further the link between *Kdm5^140^*-induced lethality and altered MAPK signaling (Drelon et al., 2019). From yeast to humans, the MAPK signaling cascade is used to regulate a myriad of cellular events in a context-dependent manner (Widmann et al., 1999; Yang et al., 2013; Eblen, 2018; Pan and O'Connor, 2021). In the prothoracic gland of *Drosophila*, the MAPK pathway is one of several signaling networks that regulates ecdysone biosynthesis (Fig. 1A). To characterize further the relationship between KDM5 and MAPK, we took a candidate-based approach by testing upstream and downstream components of this cascade for an effect on *Kdm5*-induced lethality (Fig. 1B). We used spookier-Gal4 (*spok*-Gal4) to drive expression of transgenes in a tissue-specific manner within the prothoracic gland, hereafter written as ‘*spok*>*transgene*’ (Fig. 1C) (Shimell et al., 2018; Drelon et al., 2019; Pan and O'Connor, 2021). As quantified previously, the ability of tested transgenes to mediate survival of *Kdm5^140^* animals into adulthood was calculated, and for these experiments, this survival index was normalized to that observed by *spok*-Gal4-driven expression of KDM5 (% *spok*>*Kdm5*, see Materials and Methods).

Based on the suppression of *Kdm5^140^* lethality by expression of the receptor tyrosine kinase (RTK) Torso and activated Ras (Ras^V12^) in Drelon et al. (2019), we tested whether other RTKs upstream of MAPK or the downstream kinase ERK could restore *Kdm5^140^* viability (Drelon et al., 2019). In parallel with Torso, which receives neuronal stimulation via the neurotransmitter prothoracicotropic hormone (PTTH), the RTKs anaplastic lymphoma kinase (Alk), Egfr, and PDGR and VEGF-receptor related (Pvr) can also activate MAPK signaling and impact ecdysone biosynthesis (Cruz et al., 2020; Pan and O'Connor, 2021). *spok*-Gal4-driven expression of wild-type or constitutively active (CA) forms of each of these receptors resulted in partial suppression of lethality with a mean survival index of 33.2% (Fig. 1D). Likewise, *spok*>*erk* and *spok*>*erk^CA^* resulted in survival indices of 17.4% and 41.8%, respectively (Fig. 1E). Similar to the rescue of *Kdm5^140^* by expression of KDM5 in the prothoracic gland, animals obtained through expression of RTKs or ERK were viable adult flies but with an outstretched wings phenotype and reduced lifespan (Fig. S1) (Drelon et al., 2019). Combined, these data confirm that augmenting MAPK signaling through various means of activation, not only through the Torso–Ras axis, can restore *Kdm5^140^* viability. The downstream effectors that mediate MAPK signaling in the prothoracic gland remain unknown; however, in other contexts, regulatory proteins such as Myc, the E2F1/DP heterodimer, and cell cycle mediators can be regulated by this cascade (Zhang and Liu, 2002). Because these transcription factors and cellular processes have also been associated with mammalian or *Drosophila* KDM5 function in other contexts, we next tested their ability to suppress *Kdm5^140^* lethality (Secombe et al., 2007; Benevolenskaya et al., 2005; Drelon et al., 2019). Expression of Myc, E2F1 and DP, or Cyclin E did not alter *Kdm5*-induced lethality, suggesting that other, as yet unidentified, regulators of gene expression function with KDM5 in the context of the prothoracic gland (Fig. 1F).

To determine whether this relationship with *Kdm5^140^* lethality is specific to the MAPK pathway, we examined other signaling pathways that mediate prothoracic gland function, many of which show extensive crosstalk (Fig. 1A). Specifically, we tested the IIS, SWH and TOR pathways. These three pathways are among the best characterized for their roles in the prothoracic gland, particularly in the regulation of homeostatic metabolic processes such as autophagy and lipid processing for hormone production (Danielsen et al., 2013, 2014, 2016; Texada et al., 2019). To test the IIS cascade, we expressed an activated form of the insulin receptor (*spok*>*InR^CA^*) or the downstream transcription factor Foxo (*spok*>*foxo*). Expression of InR or Foxo did result in suppression of *Kdm5^140^* lethality with survival indices of 65.6% and 24.8%, respectively (Fig. 1G). Although we previously saw no defective activation of the IIS pathway by examining phoso-Akt levels via western blot in *Kdm5^140^* animals, it is possible that ectopic insulin signaling can act on similar downstream targets or compensate in some other way for MAPK defects (Drelon et al., 2019). In contrast, SWH signaling, activated by RNA interference (RNAi) of *wts* (*spok*>*wts-RNAi* #1 and 2) or overexpression of wild-type or constitutively active *yki* transgenes (*spok*>*yki*, *spok*>*yki^CA^*) did not consistently suppress lethality (Fig. 1G). Although suppression was observed using *yki^CA^*, similar to the IIS cascade, this may indicate that some activation of these signaling pathways is able to compensate for the MAPK defects of *Kdm5^140^* animals. These results could be due to crosstalk between these pathways and/or upregulation of common targets involved in regulation of ecdysone biosynthesis and prothoracic gland function.

Prothoracic gland cells have distinct energetic and other cellular homeostatic requirements for which proper balance of TOR signaling is essential (Danielsen et al., 2016; Pan et al., 2019, 2020; Texada et al., 2019; Yamanaka, 2021). For this reason, we tested several manipulations of TOR signaling and autophagy via both activation (*spok*>*Rheb*, *spok*>*S6K*, *spok*>*TSC-RNAi*) and repression (*spok*>*TOR^DN^*). Interestingly, none of these TOR pathway manipulations affected *Kdm5^140^* lethality (Fig. 1H). Thus, although regulation of autophagy is one cellular process on which all tested signaling pathways are known to converge, the lethality of *Kdm5^140^* mutants does not appear to be caused by lack of TOR pathway regulation. Taken together, there are multiple pathways capable of suppressing *Kdm5^140^* lethality via activity in the prothoracic gland, but it is not yet clear whether these results are due to crosstalk between pathways or compensatory activation of shared downstream targets. Moreover, it remains an open question which downstream transcription factors are responsible for the cellular programs activated by this signaling that are crucial for development and adult viability.

### *Kdm5* expression is required during mid to late larval stages for viability

**Fig. 2.**
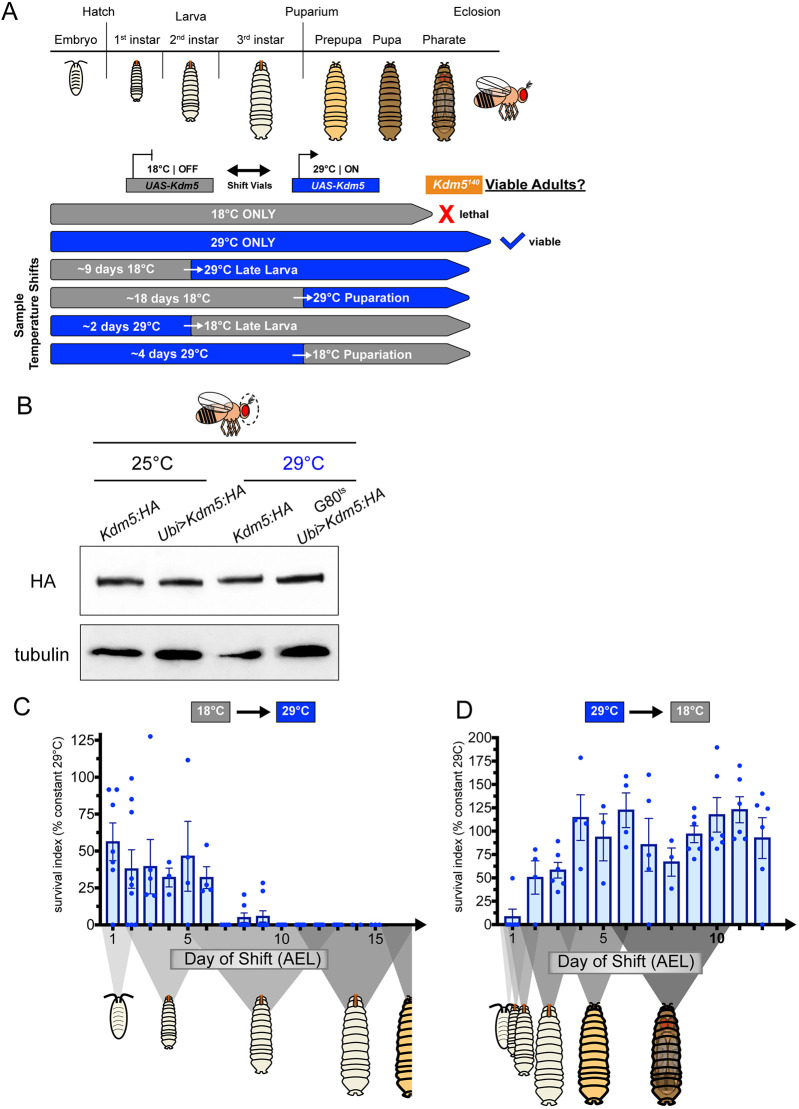
**Temporally restricted rescue of KDM5 expression reveals requirements for KDM5 in mid to late larval stages.** (A) Schematic showing example shifts between restrictive (18°C) and permissive (29°C) temperatures to constrain KDM5 expression in the *Kdm5^140^* background. (B) Western blot of adult heads showing KDM5:HA protein levels (top) from control [*Kdm5*:3xHA or *Ubi*>*Kdm5:HA* (*Kdm5^140^* background)] and temporal experiment [G80^ts^ *Ubi*>*Kdm5:H*A (*Kdm5^140^* background)] animals at standard (25°C) and experimental (29°C) temperatures. α-tubulin was used as loading control. (C) Quantification of survival index for induction of expression of KDM5 at progressively later days during development in tub-Gal80^ts^/+; *Kdm5^140^*, *Ubi*-Gal4/*Kdm5^140^*; UAS*-Kdm5:HA*/+ animals relative to that of control vials kept at constant 29°C. *x*-axis schematic demonstrates approximate developmental progression of *Kdm5^140^* animals at 18°C each day AEL. *n*=134, 216, 143, 275, 120, 124, 107, 162, 178, 218, 142, 134, 125, 111, 101, respectively. Error bars represent s.e.m. (D) Quantification of survival index for inhibition of expression of KDM5 at progressively earlier days during development (29°C to 18°C) in tub-Gal80^ts^/+; *Kdm5^140^*, *Ubi*-Gal4/*Kdm5^140^*; UAS*-Kdm5:HA*/+ animals relative to that of control vials kept at constant 29°C. *x*-axis schematic demonstrates developmental progression of *Kdm5^140^* animals at 29°C each day AEL. *n*=106, 103, 176, 111, 123, 107, 111, 117, 210, 137, 117, 133, 111, respectively. Error bars represent s.e.m.

Our targeted candidate approaches identified regulatory pathways, but not key KDM5-mediated downstream processes linked to viability. Prior to carrying out unbiased transcriptomic and genomic-binding studies, we first needed to determine when during development KDM5 is required. To do this, we ubiquitously expressed the UAS-*Kdm5* transgene using *Ubi*-Gal4 within defined windows of time during development in the *Kdm5^140^* background (Fig. 2A). To facilitate temporal activation of *Kdm5* expression, we included a transgene ubiquitously expressing temperature-sensitive Gal80 (tub-Gal80^ts^) (McGuire et al., 2003). At 18°C, the Gal80^ts^ prevents UAS-*Kdm5* transgene activation, thus *Kdm5^140^* animals with tub-Gal80^ts^, *Ubi*-Gal4 and UAS-*Kdm5* incubated at 18°C failed to reach adulthood (Fig. 2A). At 29°C, Gal80^ts^ is inactive, which allows expression of the UAS-*Kdm5* transgene, resulting in adult fly viability (Fig. 2A). At the permissive temperature of 29°C, we observed protein levels similar to both endogenous KDM5 and to our previously published system in which flies were grown at 25°C without Gal80^ts^ (Fig. 2B) (Drelon et al., 2019). Using this system, *Kdm5* expression was turned on at progressively later days during development by transferring the animals from 18°C to 29°C (Fig. 2A). The extent to which temporally restricted expression of *Kdm5* rescued viability is reported as a survival index normalized to the rescue observed by continuous expression of *Kdm5* (*Ubi*>*Kdm5* at constant 29°C, see Materials and Methods). Temperature shifting animals early in development led to robust rescue (Fig. 2C). In contrast, activating the UAS-*Kdm5* transgene in animals that had reached mid larval stages (2nd to 3rd instar) or later resulted in a failure to rescue adult viability (Fig. 2C). Thus, *Kdm5* expression is required prior to pupal stages and as early as mid to late larval stages, although we cannot yet rule out additional roles later in development. Additional complementary experiments in which UAS-*Kdm5* transgene expression was inhibited progressively later in development were also performed by shifting animals from 29°C to 18°C (Fig. 2A). These data revealed that transferring animals during earlier larval stages in development failed to rescue viability robustly, confirming key role(s) for KDM5 during the mid to late larval window of the *Drosophila* life cycle (Fig. 2D). We therefore focused subsequent experiments of KDM5 function during these stages of development.

### KDM5 directly regulates transcription of metabolic processes in the prothoracic gland

**Fig. 3.**
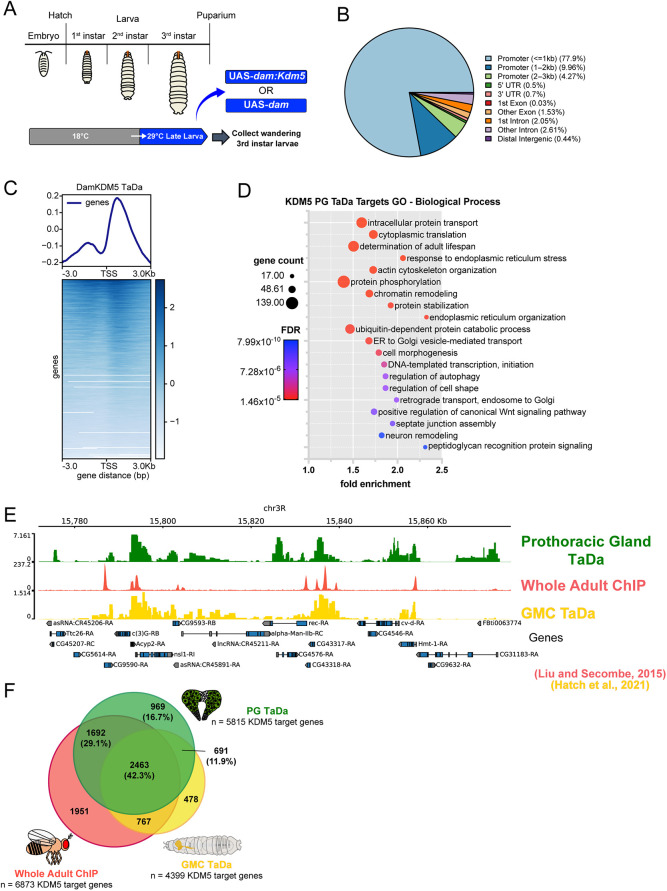
**Genome binding profiling of KDM5 by targeted DamID (TaDa) identifies conserved and tissue-specific targets.** (A) Time course of TaDa experiment, which restricted *spok*>*dam:Kdm5* or *spok*>*dam* expression to the last 48 h of larval development. *n*=100 larvae per sample, four replicates per genotype. (B) Distribution of Dam:KDM5 binding genomic regions showing enrichment for promoter-proximal regions. (C) Genomic binding localization of average Dam:KDM5 TaDa profile showing binding near the transcriptional start site (TSS). (D) GO biological process analyses of candidate KDM5 target genes identified from TaDa. Representative terms shown; see Table S3 for a full list. ER, endoplasmic reticulum. (E) Representative genome browser image showing KDM5 binding in prothoracic gland TaDa experiment juxtaposed with published data sets from whole adult KDM5 ChIP-seq and ganglion mother cell (GMC) TaDa. (F) Overlap of KDM5-bound genes across data sets. *P*<0.00001 (Fisher's exact test) for overlaps of prothoracic gland (PG) TaDa compared with whole adult ChIP and with GMC TaDa.

To investigate the roles of KDM5 in regulating gene expression programs within the prothoracic gland, we identified genomic regions bound by KDM5 in this tissue. Traditional genomic binding approaches, such as chromatin immunoprecipitation with sequencing (ChIP-seq), are limited for this small tissue that comprises only ∼50 cells. We therefore performed Targeted DamID (TaDa), which requires less input material and can be carried out with tissue- and temporal-specific resolution, to survey the genomic targets of KDM5 in these cells (Marshall and Brand, 2015, 2017; Marshall et al., 2016; Hatch et al., 2021). *spok*-Gal4 was used to drive expression of a UAS transgene encoding either a Dam:KDM5 fusion protein or Dam alone, the normalization control, in prothoracic gland cells of wild-type animals. Using tub-Gal80^ts^, expression of the TaDa transgenes was restricted to mid to late larval stages by shifting larvae from 18°C to 29°C and collecting wandering 3rd instar larvae [120-168 h after egg laying (AEL) at 18°C] (Fig. 3A). Confirming the robustness of our data, quadruplicate TaDa replicates showed a strong correlation, and an average Dam:KDM5 binding profile was used for subsequent analyses (Fig. S2). Similar to previous studies of KDM5 family proteins across species, most KDM5 binding occurred near promoter regions (Fig. 3B,C) (Lloret-Llinares et al., 2012; Beshiri et al., 2012; Liu and Secombe, 2015; Iwase et al., 2016; Hatch et al., 2021; Wang et al., 2023). This localization at or near promoters enabled us to identify nearby genes as candidate targets of KDM5 regulation.

In total, KDM5 peaks mapped to 5815 genes using a cutoff of false discovery rate (FDR)<0.01 (Table S1). Gene Ontology (GO) analyses for biological processes enriched in this gene list produced a range of terms including processes related to cellular transport, metabolism and signaling (Fig. 3D, Table S3). To assess the KDM5 binding targets in the prothoracic gland in relation to other contexts, we compared these data with existing ChIP-seq and TaDa data sets from whole adult flies and ganglion mother cells (neuronal precursors), respectively (Liu and Secombe, 2015; Hatch et al., 2021) (Fig. 3E). This revealed a significant overlap between the prothoracic gland TaDa and either dataset, with 2463 genes being bound in all three data sets (42.3% of all prothoracic gland targets) (Fig. 3F). This overlap of KDM5 targets may represent genes regulated by KDM5 across developmental stages and tissues. Overall, KDM5 appears to have the potential to regulate a large portion of the coding genome in the prothoracic gland, and KDM5 likely has both tissue-specific and universal functions.

**Fig. 4.**
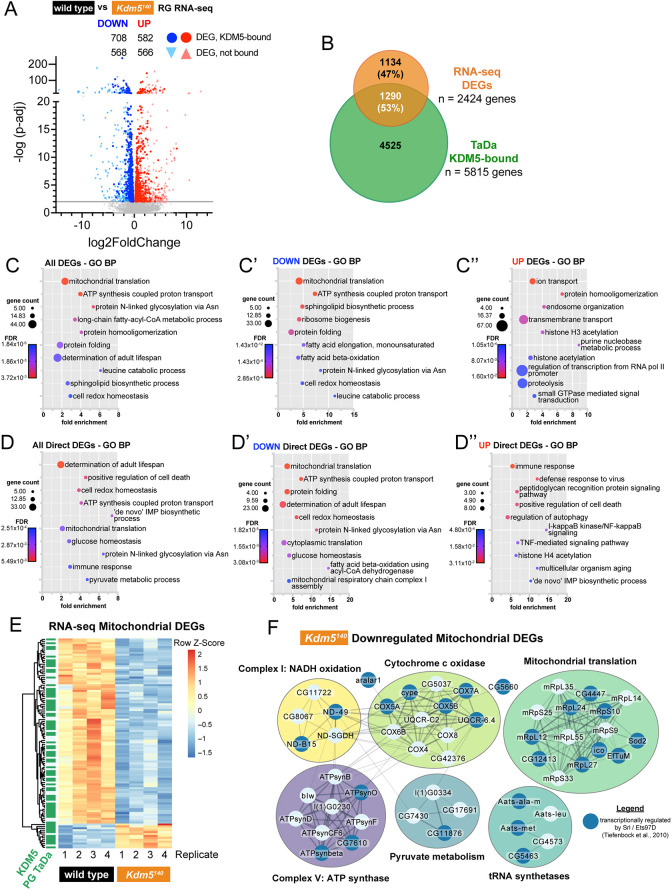
**RNA-seq reveals transcriptional dysregulation of mitochondrial genes in *Kdm5^140^* mutants.** (A) Volcano plot comparing RNA-seq of *Kdm5^140^* and wild-type ring glands. Differentially expressed genes (DEGs) with a false discovery rate (FDR)<0.01 are colored blue (downregulated) and red (upregulated), and those directly bound in KDM5 prothoracic gland TaDa are highlighted as bolded circles. *n*=80 ring glands per sample, four replicates per genotype. (B) Overlap of *Kdm5^140^* ring gland DEGs and direct KDM5 targets identified in TaDa. (C-C″) Gene Ontology Biological Process (GO BP) analyses of all DEGs (C), downregulated DEGs (C′) and upregulated DEGs (C″) using GO DAVID. Representative terms shown; see Table S3 for full lists. (D-D″) GO-BP analyses of *Kdm5^140^* DEGs that were directly bound in TaDa. Representative terms shown; see Table S3 for full lists. (E) Heatmap showing RNA-seq fragments per kilobase of transcript per million mapped reads (FPKM) of 111 genes from the mitochondrion GO term that were differentially expressed (FDR<0.01) in *Kdm5^140^* ring glands. KDM5-bound genes in TaDa are annotated (green) in left column. (F) Physical protein interaction networks of mitochondrial genes downregulated in *Kdm5^140^* ring glands. Genes regulated by both KDM5 and Srl/Ets97D are highlighted with darker blue nodes. Created with Cytoscape.

To determine the relationship between KDM5 binding and target gene expression, we performed bulk mRNA sequencing (mRNA-seq) on dissected ring glands of wild-type and *Kdm5^140^* wandering 3rd instar larvae. Similar to previous transcriptional studies, RNA-seq was carried out from dissected ring glands to assay the prothoracic gland transcriptome, as this cell type comprises the majority of the mass of the ring gland (Di Cara and King-Jones, 2016; Ou et al., 2016; Christesen et al., 2017; Nakaoka et al., 2017; Uryu et al., 2018). Using a stringent cutoff of FDR<0.01, we identified 2424 differentially expressed genes (DEGs) in *Kdm5^140^* ring glands, 1276 of which were downregulated and 1148 that were upregulated (Fig. 4A, Table S2). To determine which genes were likely to be directly regulated by KDM5, we integrated these data with the genomic binding TaDa data and found that 1290 (53.2%) of the *Kdm5^140^* DEGs had an associated KDM5 promoter peak based on the prothoracic gland TaDa (Fig. 4A,B). As seen in previous *Kdm5* mutant RNA-seq experiments, direct KDM5 targets exhibited relatively subtle changes to gene expression, and the DEGs with the largest log2 fold change appeared to be indirectly regulated by KDM5 (Fig. 4A) (Liu and Secombe, 2015; Zamurrad et al., 2018; Belalcazar et al., 2021; Hatch et al., 2021). GO analyses of all DEGs produced metabolic terms, including biological processes involving mitochondria and lipid metabolism (Fig. 4C, Table S3). The enrichment for these terms appeared to be driven by downregulated DEGs, as analysis of that subset produced many of the same GO terms, whereas that of upregulated genes featured processes involving cellular transport and chromatin dynamics (Fig. 4C′,C″, Table S3). Among the KDM5-bound DEGs, there was a similar trend with the top GO analysis terms related to mitochondrial processes and cellular respiration (Fig. 4D-D″, Table S3). These genome binding and transcriptomic analyses reveal that gene expression programs under the direct regulation of KDM5 span various cellular processes in the prothoracic gland, particularly those involving metabolism and mitochondria.

### KDM5-regulated transcription is developmentally required for proper mitochondrial dynamics

KDM5 proteins have been previously associated with mitochondrial activity in *Drosophila* and in mammalian cells, although the mechanisms and biological implications of these connections remain unclear (Liu and Secombe, 2015; Varaljai et al., 2015; Liu et al., 2023). Within the GO database, 353 genes are classified in the mitochondrion biological processes category, and, of these, 111 genes were differentially expressed in *Kdm5^140^* animals. Most of these genes were both downregulated in *Kdm5^140^* across our RNA-seq replicates and directly bound in the TaDa data (Fig. 4E). Investigation of known physical interactions within this downregulated mitochondrial gene set identified connections including components of cytochrome c oxidase and ATP synthase complexes as well as mitochondrial translation (Fig. 4F). Combined, these data suggest that KDM5 is needed to maintain the expression of genes essential to mitochondrial biology, which could contribute to its essential developmental activities.

**Fig. 5.**
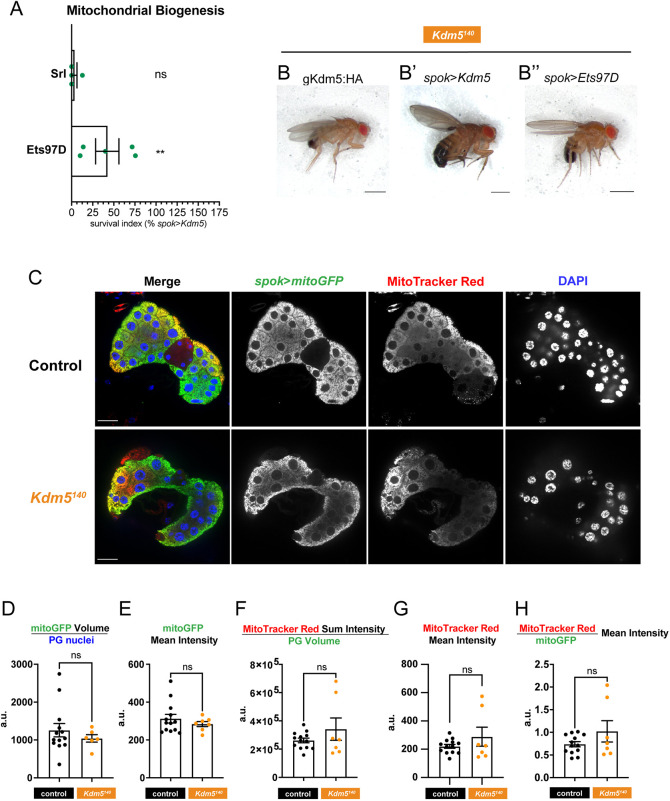
**KDM5 regulates mitochondrial dynamics in the prothoracic gland that are crucial for development.** (A) Quantification of survival index for expression of mitochondrial biogenesis factors in *Kdm5^140^* background relative to *spok*>*Kdm5*. *n*=922, 757, respectively. **P<0.01; ns, not significant (Fisher's exact test compared with ‘no UAS’ control). Error bars represent s.e.m. (B-B″) *Kdm5^140^* adult flies with lethality suppressed by genomic region *Kdm5:HA* transgene (B), *spok*>*Kdm5* (B′) or *spok*>*Ets97D* (B″). Scale bars: 750 μm. (C) Representative single *z*-slices of larval ring glands expressing *spok*>*mitoGFP* and stained with MitoTracker Red. Control genotype: *Kdm5^140^*/CyO-GFP. Scale bars: 20 μm. (D-H) Quantification of mitochondrial reporters across prothoracic gland samples. a.u., arbitrary units. *n*=13, 7, respectively. ns, not significant (Wilcoxon rank sum test). Error bars represent s.e.m. (D) Quantification of total mitoGFP signal volume per prothoracic gland (PG) nucleus. (E) Quantification of mean mitoGFP signal intensity. (F) Quantification of total MitoTracker Red signal sum intensity per gland volume. (G) Quantification of mean MitoTracker Red signal intensity. (H) Quantification of the ratio of mean intensity of MitoTracker Red over mitoGFP signal. Images are representative of 13 control and 7 *Kdm5^140^* samples.

The energetic demands of polyploid prothoracic gland cells may make this cell type particularly sensitive to perturbations in mitochondrial activity. In addition to generating key cellular metabolites, mitochondria in the prothoracic gland are important sites for Halloween gene (ecdysone biosynthetic enzymes) activity in processing stored lipid precursors for hormone production (Sandoval et al., 2014; Jacobs et al., 2020; Pan et al., 2020). To test whether the gene expression changes associated with mitochondrial function were linked to the lethality caused by loss of *Kdm5*, we sought genetic approaches to attenuate this deficit. Spargel (Srl) and Ets97D, homologous to mammalian PGC1α and GABPα/NRF-2, respectively, are transcriptional activators known to regulate the expression of mitochondrial biosynthesis genes (Tiefenbock et al., 2010; Tain et al., 2017; Sainz de la Maza et al., 2022). Previously published microarray experiments showed that Srl and/or Ets97D regulate many of the mitochondrial genes found to be downregulated in *Kdm5^140^* animals (Fig. 4F, highlighted in darker blue) (Tiefenbock et al., 2010). In light of these transcriptional data, we tested whether expression of Srl or Ets97D in the prothoracic gland could restore viability to *Kdm5^140^* animals. Whereas *spok*>*srl* failed to suppress *Kdm5^140^* lethality, expression of Ets97D did restore viability to produce morphologically normal adult flies (Fig. 5A-B″). KDM5-mediated activation of mitochondrial function genes in the prothoracic gland are therefore likely to be necessary for animal survival.

To assess whether *Kdm5^140^* animals exhibited visible mitochondrial phenotypes, we expressed a UAS-*mitoGFP* reporter with *spok*-Gal4 to examine mitochondrial networks (Fig. 5C). Assaying overall mitochondrial mass by quantifying the mitoGFP signal volume and mean intensity per cell revealed no differences between *Kdm5^140^* and control animals (Fig. 5D,E). To assess mitochondrial energetics, we stained with MitoTracker Red, a reagent that is retained in the mitochondrial matrix of active mitochondria where the membrane is hyperpolarized (Wong et al., 2020). Similar to mitoGFP, the MitoTracker Red signal showed no significant changes in terms of sum intensity per prothoracic gland cell nor mean intensity in *Kdm5^140^* animals compared with controls (Fig. 5F,G). Furthermore, the ratio of MitoTracker Red/mitoGFP signal, an analysis of overall mitochondrial activity per mass, was not different between genotypes (Fig. 5H). Thus, there were no observable changes to mitochondrial abundance or energetics at a tissue-wide level.

**Fig. 6.**
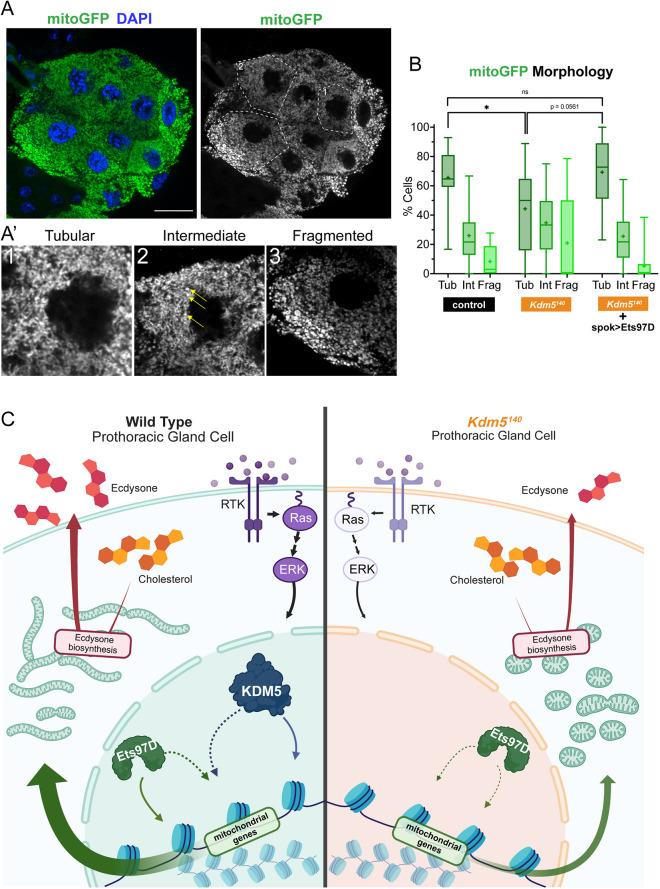
**Model for KDM5-mediated transcriptional regulation of mitochondrial biology in prothoracic gland cells.** (A,A′) Representative single *z*-slice of larval ring gland expressing *spok*>*mitoGFP*. Dashed lines delineate representative cells in A′. (A′) High-magnification images of representative cells of each morphological classification. Yellow arrows indicate fragmented mitochondria within an intermediate cell. Scale bar: 20 μm. (B) Quantification of mitoGFP morphological classifications normalized to total number of cells. *n*=17, 9, respectively. *P<0.05; ns, not significant (nonparametric unpaired *t*-test). Box limits represent 25th-75th percentiles, horizontal line median and whiskers minimum and maximum values. (C) In *Kdm5*^*140*^ mutants, prothoracic gland cells exhibit defects in MAPK signaling and mitochondrial gene expression and morphology, highlighting roles for KDM5 in regulating transcriptional programs that coordinate these processes crucial to ecdysone dynamics and proper development.

Focusing our analysis to the cellular scale, we examined the morphology of the mitoGFP-marked mitochondrial networks, classifying them as tubular, intermediate and fragmented, similar to previous studies (Fig. 6A,A′) (Deng et al., 2015; Kashatus et al., 2015). Control animals displayed a majority of tubular cells with elongated and highly branched mitochondria (Fig. 6B). In contrast, *Kdm5^140^* prothoracic glands showed a significant decrease in the proportion of cells with tubular morphology, with these glands featuring more rounded and isolated mitochondrial populations of the intermediate and fragmented classifications. These results indicate that, although there are no changes to overall abundance, mitochondrial biology is disrupted at the organelle level in *Kdm5^140^* mutants. Similar analyses in larval fat body cells using *CG*-Gal4>*mitoGFP* revealed decreased elongation and increased fragmentation in mitochondria of *Kdm5^140^* animals (Fig. S3). Thus KDM5-mediated regulation of mitochondrial function is not limited to the prothoracic gland, although this role does appear to be particularly important in this cell type. The increase in fragmented mitochondria in *Kdm5^140^* could be due to defect(s) in a range of processes, including altered mitochondrial dynamics or altered functioning due to the stress response. Given that the mitochondrial biosynthesis regulator Ets97D can suppress *Kdm5^140^* lethality via *spok*>*Ets97D*, we examined the mitochondrial networks of these animals and observed suppression of the morphological changes to prothoracic gland cell mitochondria in *Kdm5^140^* (Fig. 6B). Taken together, our data show that KDM5 transcriptional regulation in prothoracic gland cells is needed for mitochondrial homeostasis, and defects in mitochondria and cellular respiration in the prothoracic gland are key contributors to the lethality caused by loss of KDM5 (Fig. 6C).

## DISCUSSION

In this study, we incorporated unbiased genome-wide data with targeted genetic and cellular analyses to expand our understanding of how KDM5-regulated transcriptional programs regulate crucial cellular processes during development. Although KDM5 is important across many cell types, we focused on the prothoracic gland, where KDM5 is important for survival (Drelon et al., 2019). This work has revealed important roles for KDM5 with respect to intracellular signaling and processes, notably MAPK pathway regulation and mitochondrial homeostasis. Consistent with our prior observation that loss of KDM5 resulted in reduced MAPK signaling (Drelon et al., 2019), prothoracic gland-specific expression of MAPK-activating RTKs or ERK suppressed *Kdm5^140^* lethality. Despite the energy-regulatory pathway of autophagy being one of the best characterized cellular processes downstream of signaling pathways in the prothoracic gland, enhancing or attenuating this process had no effect on the lethality of *Kdm5^140^*. Instead, our KDM5 genomic binding and gene expression analyses pointed to a vital role for KDM5 in the regulation of a range of metabolic processes needed for cellular homeostasis, particularly mitochondrial function. Confirming the importance of KDM5-regulated expression of genes that support mitochondrial activity, we observed morphological changes to these organelles. Moreover, these changes are likely to be important for the essential functions of KDM5, as prothoracic gland expression of the transcription factor Ets97D, a known regulator of genes needed for mitochondrial function, suppressed the morphological defects and lethality of *Kdm5^140^* animals.

Associations between KDM5 proteins and mitochondrial gene regulation have been previously found in adult flies as well as in cultured mammalian cells with KDM5A/RBP2 and KDM5C (Lopez-Bigas et al., 2008; Peng et al., 2009; Liu and Secombe, 2015; Varaljai et al., 2015; Kim et al., 2022; Liu et al., 2023). Muscle cells of hypomorphic *Kdm5* adult flies showed abnormal mitochondrial shape, altered expression of redox-related genes, and increased sensitivity to oxidative stress (Liu et al., 2014; Liu and Secombe, 2015). Interestingly, most of these changes to mitochondrial gene expression in adult flies do not overlap with those in the prothoracic gland, which were linked to respiratory chain complexes and translation. KDM5 is therefore likely to play different roles in distinct cell types. In human cells, the KDM5-mitochondria relationship has primarily been examined during differentiation. Although we observed KDM5 to be required for the activation of mitochondrial genes, in myogenic precursor and promonocytic cells, KDM5A represses mitochondrial genes (Varaljai et al., 2015; Lopez-Bigas et al., 2008). Consistent with the disparate changes to transcription, inhibition of human KDM5A led to more dense tubular mitochondrial networks, whereas we observed mitochondrial fragmentation in *Drosophila* prothoracic gland and fat body cells lacking all KDM5 function. In findings more similar to our data, *Kdm5c*-deficient mouse monocytes and osteoclasts have decreased mitochondrial gene expression resulting in decreased bioenergetic metabolism (Liu et al., 2023). Therefore, KDM5 proteins regulate the transcription of genes integral to mitochondrial function, but it is possible that whether this results in increased or decreased expression depends on the energy demands of a given cell type and/or the developmental cellular context. Indeed, it is notable that in muscle cell differentiation, KDM5A appears to function as part of an E2F/DP/pRb axis to regulate mitochondrial function in myogenic precursor cells, but we find that E2F1/DP does not suppress *Kdm5^140^* lethality (Varaljai et al., 2015). Integrating these studies across species, it is apparent that mitochondrial and other metabolic genes are conserved targets of KDM5-mediated transcriptional regulation, but specific mechanisms of KDM5-mediated regulation may be elicited by differing cellular conditions. Notably, prothoracic gland and fat body cells are terminally differentiated and polyploid, requiring different homeostatic dynamics than the differentiating precursor cells of the mammalian studies. This may lead to KDM5 interacting with distinct gene regulatory complexes, or possibly employing histone demethylase-dependent and -independent activities to alter transcription. Indeed, based on our observation that flies lacking KDM5-mediated histone demethylase activity are viable, the regulation of mitochondrial-related genes in the prothoracic gland is likely to be independent of its enzymatic function (Drelon et al., 2019).

The transcriptional regulators Ets97D and Srl are involved in the activation of many of the same genes required for mitochondrial function that are regulated by KDM5 (Tiefenbock et al., 2010). Although transgenic expression of Ets97D can compensate for the loss of KDM5 in the prothoracic gland, it is not clear whether *Kdm5^140^* animals die as a result of reduced Ets97D activity, as its expression was not decreased in our RNA-seq data (Table S2). Additionally, *spok*>*srl* failed suppress *Kdm5^140^* lethality. These results may reflect differences in the function of these proteins or variation in regulation at the post-translational modification level. As both Ets97D and Srl are known to be regulated in this manner, *Kdm5^140^*-dependent changes in factors upstream of Ets97D and Srl-dependent transcription may be responsible for the phenotypes found in this study (Sunesen et al., 2003; Charlot et al., 2010; Tain et al., 2017; Luo et al., 2019).

The simplest synthesis of our *Kdm5^140^* experiments is that KDM5 is needed for proper activation of the MAPK pathway and that this alters the activation of genes related to mitochondrial function, possibly through Ets97D. The Ets family transcription factors are well-documented targets of MAPK signaling regulation, and current data for the mammalian GABPα, although mixed, suggests that this mechanisms could exist for Ets97D in flies (Foulds et al., 2004; Rosmarin et al., 2004; Selvaraj et al., 2015). Similar to Ets97D, our RNA-seq data did not reveal notable changes to components of MAPK signaling; thus, it remains unknown how KDM5 regulates this pathway. The MAPK cascade inputs into many processes across the cell, impacting metabolism through a variety of levels of regulation. Although the relationships between MAPK and metabolic processes such as autophagy and glycolysis are more established, direct connections between MAPK signals and mitochondrial biology have been documented (Galli et al., 2009; Haq et al., 2013; Javadov et al., 2014; Kashatus et al., 2015). In fact, most of the existing links between MAPK and mitochondria have been identified in the context of cancer cells and RASopathy developmental disorders. Mitochondrial dynamics can be altered in various cancers, and some studies have looked at mitochondria as a potential target to antagonize MAPK-driven tumors (Serasinghe et al., 2015; Corazao-Rozas et al., 2016; Marchetti et al., 2018; Ferraz et al., 2020). Furthermore, RASopathies, a collection of rare diseases driven by germline MAPK mutations, exhibit forms of mitochondrial dysfunction that contribute to bioenergetic defects (Dard et al., 2018; Kontaridis and Chennappan, 2022). In both cancer and neurodevelopmental disorders, KDM5 proteins may be involved in the regulation of this axis of MAPK-mediated metabolic changes. The potential role for KDM5 with both MAPK signaling and mitochondrial regulation indicates that KDM5 could be considered as a target when treating these disorders.

One outstanding question from these studies is how KDM5-regulated transcriptional programs within the prothoracic gland mediate survival at an organismal level. Anoar et al. (2021) hypothesize that neurons are particularly susceptible to mitochondrial defects because of high energetic demands and because, as long-lived post-mitotic cells, they cannot dilute out defective organelles by cell division (Anoar et al., 2021). Similarly, prothoracic gland cells exit the cell cycle at the embryonic stage and must survive as large, polyploid cells with bioenergetic requirements into the pupal stages to coordinate *Drosophila* developmental programs. KDM5-mediated mitochondrial regulation may be a key facet in the life cycle of the prothoracic gland cells in maintaining the metabolic homeostasis needed to regulate the production of the ecdysone hormone. Although *Kdm5^140^* mutants show lower levels of ecdysone that delay development, they still undergo metamorphosis to form adult structures and therefore must have sufficient ecdysone to facilitate this, whether that is through prothoracic gland function and/or other mechanisms, such as recycling stored ecdysone (Drelon et al., 2019; Scanlan et al., 2023). Although *Kdm5^140^* animals can stimulate gross adult structure formation, some of the finer details of the underlying tissue, particularly synapse formation between neurons in the brain and into peripheral tissues, may depend on the quantity of ecdysone hormone and the specific timing of ecdysone pulses. During metamorphosis, the neuronal networks across the animal undergo significant growth, pruning and synapse formation for innervation across the newly formed adult body (Truman and Riddiford, 2023). This neuronal patterning is coordinated in part by ecdysone-responsive transcriptional elements, and likely hinges on proper timing for synaptic inputs and outputs to meet appropriately (Oliveira and Homem, 2023). Overcoming the *Kdm5*-dependent defects by transgene-mediated modulation of mitochondrial dynamics may restore prothoracic gland cell homeostasis and function sufficiently for the ecdysone production and release program to successfully guide this neuronal remodeling that needs to occur in pupae. Future studies analyzing the relationship between KDM5-regulated mechanisms, ecdysone temporal dynamics, and mitochondrial homeostasis in the prothoracic gland will be key in defining these essential developmental programs.

## MATERIALS AND METHODS

### Key resources

See Table S4 for a list of key resources.

### Fly husbandry

All flies were kept at 25°C on standard food at 50% humidity and a 12 h light/dark cycle unless otherwise stated. Food (per liter) contained 18 g yeast, 22 g molasses, 80 g malt extract, 9 g agar, 65 g cornmeal, 2.3 g methyl para-benzoic acid and 6.35 ml propionic acid. For studies comparing wild-type and *Kdm5^140^* mutant larvae, animals were matched for developmental stage, not chronological age, as we have done previously (Drelon et al., 2018, 2019; Belalcazar et al., 2021; Hatch et al., 2021). Thus, at 25°C, control wandering 3rd instar larvae were collected ∼120 h AEL, and *Kdm5^140^* larvae at the same stage were collected ∼168 h AEL. For all analyses, we used equal numbers of male and female animals and pooled data given that we did not observe any sex-specific effects. In all experiments testing suppression of *Kdm5^140^* lethality, vials resulting in *n*<12 or *n*>80 total eclosed adult flies were excluded from final analyses. This vial density was experimentally determined to be optimal for potential survival of *Kdm5^140^* animals as under- or overcrowding outside this density introduced additional variables, including inconsistent food conditions and larval competition with control CyO-GFP (heterozygous) animals.

### Fly strains and genetics

A detailed list of the genotypes of the flies used in each figure is given in Table S5.

The *Kdm5^140^* mutant allele, *Kdm5:3xHA*, *UASp-Kdm5:HA*, *UAS-LT3-dam:Kdm5*, and genomic region *Kdm5:HA* transgenes have previously been described (Drelon et al., 2018; Hatch et al., 2021; Navarro-Costa et al., 2016). The *spok*-Gal4, UAS-*torso*, UAS-*Alk* and UAS-*Alk^CA^* lines were kindly shared by Michael O'Connor (University of Minnesota, MN, USA). The UAS-*srl* line was kindly shared by Grace Zhai (University of Miami, FL, USA) with permission from Christian Frei (University of Zurich, Switzerland). The UAS-*Ets97D* line was kindly shared by Martine Simonelig (Institut de Genetique Humaine, France) with permission from Christian Frei. The UAS-*LT3-dam* line was kindly shared by Andrea Brand (The Gurdon Institute, University of Cambridge, UK). All other strains were obtained from the Bloomington *Drosophila* Stock Center (see Table S4).

### Immunohistochemistry

Wandering 3rd instar larval brain-ring gland complexes were dissected in ice cold 1× PBS and fixed in 4% paraformaldehyde (PFA) in PBS at room temperature for 20 min. Samples were washed three times in 1× PBST (PBS+0.1% Triton X-100) for 10 min each. Brain-ring gland complexes were transferred to 0.5 μl tubes for blocking in 1× PBST+5% normal donkey serum (NDS) for 30 min, followed by primary antibody incubation overnight while rotating at 4°C. After three 15 min washes in 1× PBST, samples were incubated in secondary antibodies at room temperature rotating for 2 h. Samples were then washed three times in 1× PBST and ring glands were dissected from brain tissue in ice-cold 1× PBS. Finally, ring glands were mounted with Fluoromount-G DAPI (Southern Biotech), and slides were stored at 4°C for imaging within 1-3 days.

A similar protocol was followed for mitochondrial immunostaining with the following exceptions. Larval brain-ring gland complexes were dissected in ice cold 1× Schneider's Medium (Gibco, Thermo Fisher Scientific), and then incubated in 500 nM MitoTracker Red CMXRos (Invitrogen, diluted in 1× Schneider's Medium) for 30 min protected from light. After two washes in 1× PBS, samples were fixed in 4% PFA in PBS. Additionally, after secondary antibody incubation, samples were washed five times in 1× PBST prior to mounting. For larval fat body, the same dissection protocol was followed with wandering 3rd instar larvae, except the samples were fixed, washed, and immediately mounted.

The following primary antibodies were used: mouse anti-HA (1:100; 2367, Cell Signaling Technology) and rabbit anti-GFP (1:100; A11122, Invitrogen). Primary antibodies were prepared in 5% NDS in PBST. The following secondary antibodies were used: goat anti-mouse Alexa Fluor 568 (1:500; A11004, Thermo Fisher Scientific) and goat anti-rabbit Alexa Fluor 488 (1:500; A11034, Thermo Fisher Scientific). Secondary antibodies were prepared in 5% NDS in PBST.

### Image acquisition and processing

Schematic images of prothoracic gland signaling pathways, pupal brain, and the model of KDM5 function in the prothoracic gland were created with BioRender.com. All dissected tissue images were taken on a Nikon CSU-W1 Spinning Disk confocal microscope using a 100× immersion lens (NA=1.45 oil) and 0.2 μm *z*-step size. Adult fly images were obtained using a stereomicroscope Carl Zeiss Stereo Discovery V12 with 12.5× magnification and captured using AxioVision Release 4.8 software. All images were processed with ImageJ. All Venn diagrams were generated using the R package BioVenn (v1.1.3) (Hulsen et al., 2008). Figures were composed using Adobe Illustrator.

### *Kdm5^140^* lethality suppression experiments

To identify signaling pathway components that suppressed *Kdm5^140^* lethality, *Kdm5^140^*/CyO-GFP;*spok*-Gal4 flies were crossed with *Kdm5^140^* flies carrying a UAS transgene and allowed to lay eggs for 48 h at 25°C. Animals were kept at 25°C, and all eclosed adults were scored. Using Mendelian ratios, we estimated the number of *Kdm5^140^* animals expected in each cross based on the total internal control (CyO-GFP) adults eclosed as done previously (Drelon et al., 2019). The survival index was calculated as a percentage of the total viable (lethality-suppressed) *Kdm5^140^* adults eclosed over the estimated number of *Kdm5^140^* animals in the cross. Graphed survival index data points represent biological replicate crosses normalized to the positive control *spok*>*Kdm5* rescue.

### Western blotting

For each sample, three male and three female adult heads (age 1-3 days) were homogenized in PBS, denatured in 1× loading buffer (3× Laemmli sample buffer containing 187.5 mM Tris, 6% SDS, 30% glycerol, 0.03% Bromophenol Blue and 10% β-mercaptoethanol) at 95°C for 5 min, run on a 6% 1.5 mm gel, and transferred to a PVDF membrane. The following primary antibodies were used: mouse anti-HA (1:2000; 2367, Cell Signaling Technology) and mouse anti-αTubulin (1:10,000; 12G10, Developmental Studies Hybridoma Bank). Secondary antibody used was rabbit anti-mouse (1:1000; 65-6520, Invitrogen). Blots were scanned and processed using a Kwik Quant Imager (Kindle Biosciences) scanner.

### KDM5 temporal experiments

To identify the developmental windows requiring *Kdm5* expression, *Kdm5^140^*,*Ubi*-Gal4/CyO-GFP flies were crossed with tub-Gal80^ts^,*Kdm5^140^*/CyO-GFP;UAS*-Kdm5:HA* flies and allowed to lay eggs for ∼12 h at either 18°C or 29°C. Animals raised at 18°C were transferred to 29°C to induce the expression of the *Kdm5* transgene, and all eclosed adult flies were scored. Conversely, animals raised at 29°C were transferred to 18°C to repress the expression of the *Kdm5* transgene, and adults were scored in the same way. For 18°C to 29°C shifts, days 1-15 were tested with *n*>100 flies eclosed for each day of shift. For 29°C to 18°C shifts, days 1-12 were tested in the same manner. The survival indices for these crosses were calculated in the same method as the *Kdm5^140^* lethality suppression experiments. Graphed survival index data points represent vial replicates normalized to the positive control *Ubi*>*Kdm5* at constant 29°C rescue.

### Targeted DamID and analyses

To profile the genomic regions bound by KDM5 in prothoracic gland cells, tub-Gal80^ts^;*spok*-Gal4 flies were crossed with flies carrying *UAS-LT3-dam* or *UAS-LT3-dam:Kdm5* and allowed to lay eggs for 24 h at 18°C. Animals were kept at 18°C for 5 days then transferred to 29°C for 2 days to induce the expression of the transgenes. Wandering 3rd instar larvae were collected, flash-frozen on dry ice, and stored at −80°C.

Tissue processing was performed as previously described with the following modifications (Marshall et al., 2016). TaDa was performed in quadruplicate with replicates of 100 larvae that were homogenized and digested in Proteinase K in samples of 50 larvae then pooled into replicates of 100 larvae prior to DNA extraction. Larvae were homogenized in 75 μl UltraPure Distilled Water and 20 μl 500 mM EDTA then digested with Proteinase K for 1.5 h. DNA extraction was performed using the Zymo Quick-DNA Miniprep Plus Kit. DpnI digestion, PCR adaptor ligation, DpnII digestion, and PCR amplification were performed as described. DNA was sonicated using a Diagenode Bioruptor Pico for 6 cycles (30 s on/90 s off at 4°C), and DNA fragments were analyzed using an Agilent Bioanalyzer to confirm ∼300 bp fragment size. DamID adaptor removal and DNA cleanup were performed as previously described, and samples were submitted to BGI Genomics for library construction and sequencing.

Libraries were prepared at BGI Genomics following a ChIP-seq workflow. DNA fragments were first end-repaired and dA-tailed using End Repair and A-Tailing (ERAT) enzyme. Adaptors were then ligated for sequencing and ligated DNA purified using AMPure beads. DNA was then PCR amplified with BGI primers (see Table S4) for eight cycles and PCR purified with AMPure beads. DNA was then homogenized, circularized, digested, and again purified. DNA was then prepared into proprietary DNA nanoballs (DNB™) for sequencing on a DNBSEQ-G400 platform with 50 bp single-end read length and 20 M clean reads passing filter.

For targeted DamID analyses, sequencing data were aligned to the Dm6 *D. melanogaster* genome and processed using damidseq_pipeline as previously described (Marshall and Brand, 2015, 2017; Marshall et al., 2016a). After converting to bedgraphs via damidseq_pipeline, peaks were called using find_peaks (using the parameters fdr=0.01, min_quant=.9) on the averaged replicates, and genes overlapping peaks identified using peaks2genes (Marshall et al., 2016; https://github.com/owenjm/find_peaks).

For genome localization analyses, the R package ChIPseeker (v1.34.1) was used with the average KDM5 binding BED file to generate profiles (Wang et al., 2022). GO enrichment analysis of KDM5-bound genes (FDR<0.01) utilized GO DAVID database (v2021), specifically annotation GOTERM_BP_DIRECT (Sherman et al., 2022). Genome browser image was generated using pyGenomeTracks (v3.8) utilizing BedGraph or bigWig files from: adult fly KDM5 ChIP-seq (SRX1084165) and larval neuronal precursor KDM5 TaDa (GSE166116) (Lopez-Delisle et al., 2020).

### RNA-seq

RNA-seq was carried out on pooled ring glands dissected from control (*w^1118^*) and *Kdm5^140^* wandering 3rd instar larvae. Ring glands were dissected and washed three times in ice-cold 1× PBS, transferred to TRIzol, flash-frozen on dry ice, and stored at −80°C. Eighty dissected ring glands were pooled to form each of the four replicates. Total RNA was isolated with TRIzol and Phasemaker tubes (Invitrogen), and quality was assessed by Agilent Bioanalyzer before sending to Novogene for library construction and sequencing. mRNA was purified from total RNA using poly-T oligo-attached magnetic beads. After mRNA fragmentation, first-strand cDNA and second-strand cDNA were synthesized, and cDNA fragments were purified with AMPure XP system to select for suitable sizes for PCR amplification. Library quality was assessed on the Agilent Bioanalyzer 2100 system. Libraries were sequenced on the Ilumina NovaSeq PE150 platform (2×150 bp cycles). Alignment of raw reads to the reference genome (dm6) was performed using HISAT2 (v2.0.5) for mapping, assembly via StringTie (v1.3.3b), quantification via featureCounts (v1.5.0-p3), normalized, and differential expression was determined with the DESeq2 package (1.20.0) (Pertea et al., 2016; Love et al., 2014; Liao et al., 2013).

GO enrichment analysis of protein-coding genes found to be dysregulated in *Kdm5^140^* RNA-seq data (1% FDR cutoff) was carried out using GO DAVID annotation GOTERM_BP_DIRECT (Sherman et al., 2022). The heatmap was generated using the R package pheatmap (v1.0.12) (https://CRAN.R-project.org/package=pheatmap). Physical interaction networks were determined using String and visualized using Cytoscape (v3.9.1) (Shannon et al., 2003). Single nodes without physical connection edges were excluded from the interaction network image, and Srl/Et97D regulation was identified in microarray data from Tiefenbock et al. (2010).

### Quantification and statistical analyses

All experiments were carried out in biological triplicate (minimum) and numbers (*n*) are provided for each experiment in the figure legends.

For *Kdm5^140^* lethality suppression experiments, a Fisher's exact test was performed in R Studio (v2023.03.0) comparing the survival index of each genotype with the ‘no UAS’ control genotype as done previously (Drelon et al., 2019; http://www.rstudio.com/) (****P<0.0001, ***P<0.001, **P<0.01, *P<0.05; ns, not significant). For KDM5 binding Venn diagram overlap, a Fisher's exact test was performed in R Studio.

For prothoracic gland mitoGFP and MitoTracker Red fluorescent images, the control genotype used was *Kdm5^140^*/CyO-GFP heterozygous animals that developed from the same cross alongside the *Kdm5^140^* homozygous animals because we have not seen the same developmental and lethality phenotypes from these animals (Drelon et al., 2019). Volocity software was used to quantify the intensity and three-dimensional volume of the fluorescent signal in each channel. Unpaired, two-tailed Student's *t*-test comparing control and *Kdm5^140^* genotypes was performed in GraphPad Prism (v9.5.1). mitoGFP morphological quantifications were performed as follows. For all analyses, the operator was unaware of the genotype for each image. Images were analyzed at two *z*-slice locations positioned 33% and 66% through the full *z*-plane of the sample. At each *z*-slice, all cells with nuclei clearly visible by DAPI signal at that *z*-position were identified and classified for mitochondrial morphology of tubular, intermediate or fragmented by scrolling through the *z*-slices occupied by each identified cell. Tubular morphology consisted of zero visible fragmented round mitochondria, intermediate morphology consisted of primarily tubular morphology with >1 visible fragmented mitochondria, and fragmented morphology consisted primarily of fragmented mitochondria. The proportion of cells with each morphological classification was calculated per sample (individual prothoracic gland), and a parametric unpaired *t*-test was performed in GraphPad Prism comparing each morphological classification between control and *Kdm5^140^* animals.

A similar protocol was followed for fat body mitoGFP fluorescent images with the following exceptions. In all images, all clearly visible nuclei and/or cell borders were identified, and any images not meeting these criteria for distinguishing cells were excluded. The area directly surrounding the nucleus of each fat body cell was classified with either elongated or fragmented mitochondrial morphology. The tubular and intermediate classifications were combined into an elongated classification because of the wide variation of elongated mitochondria from shorter, rod-like shapes to longer tubules, but with few clear tubular networks visible around the high concentration of lipid droplets.

## Supplementary Material

10.1242/develop.202024_sup1 Supplementary information

Table S1. TaDa-identified KDM5 target genes.

Table S2. RNA-seq from kdm5[140] ring glands.

Table S3. full list of genes identified by GO DAVID Gene Ontology.

Table S4. Key resources

Table S5. Fly genotypes
