# Supplementary material for: KDM5-mediated activation of genes required for mitochondrial biology is necessary for viability in Drosophila
Source: Development. 2023 Nov 6;150(21):dev202024. doi: 10.1242/dev.202024 (PMC10651110; doi:10.1242/dev.202024)
Supplement: Supplementary information [file develop-150-202024-s1.pdf]

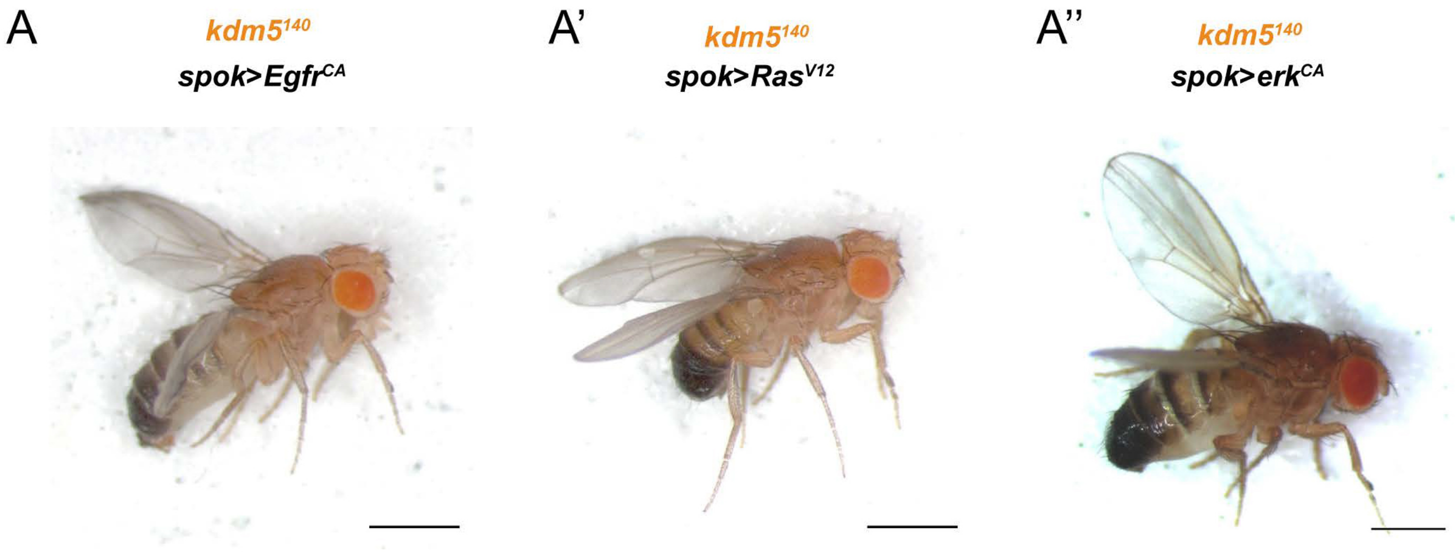

Fig. S1. ***kdm5***$^{\mathit{140}}$ **adults with lethality suppressed by MAPK components.**

(A-A'') Representative images of *kdm5*^{140} adult flies with lethality suppressed by *spok>Egfr*^{CA} (A), *spok>Ras*^{V12} (A'), or *spok>erk*^{CA} (A''). Scale bars: 750 µm.

Development • Supplementary information

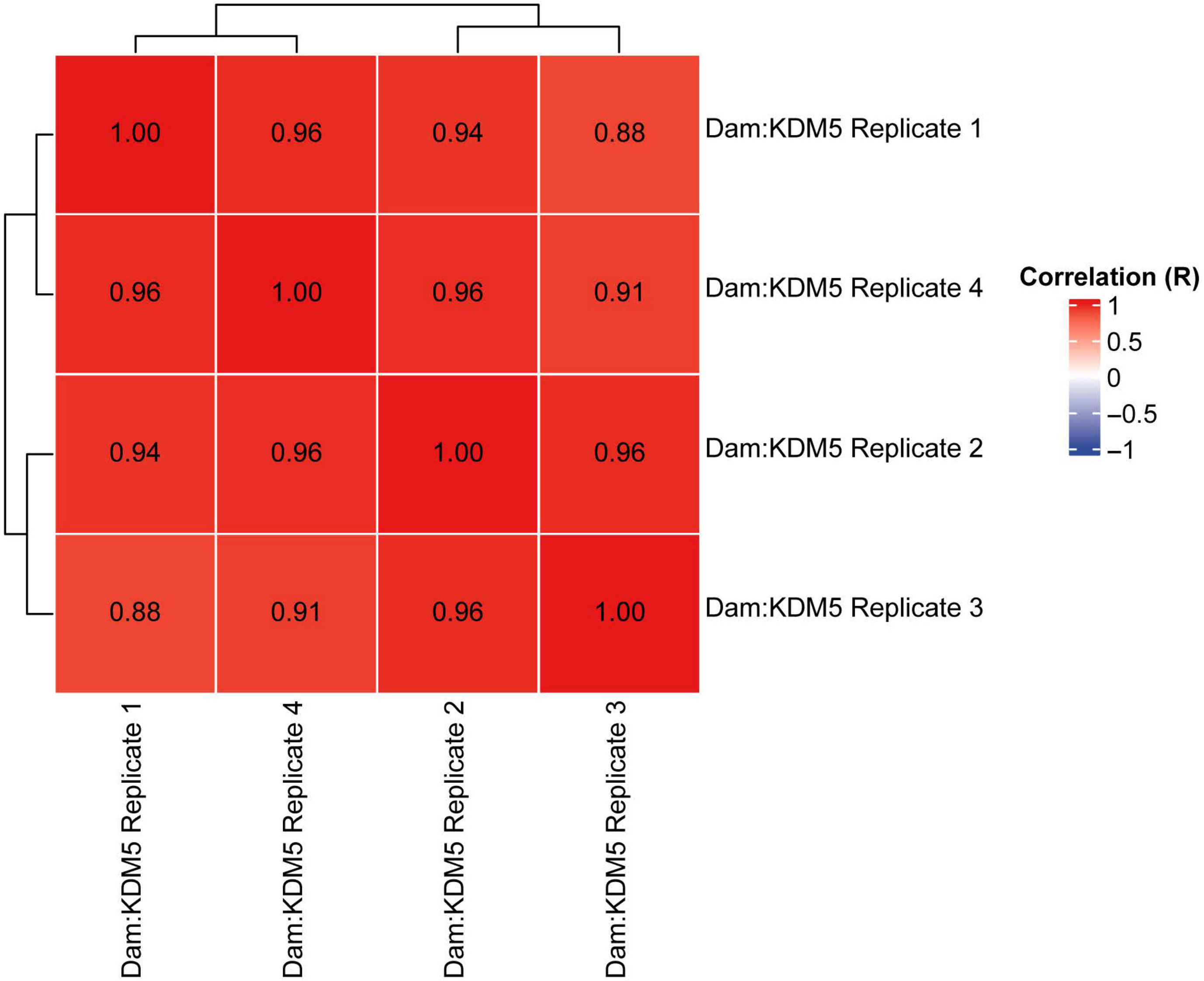

Fig. S2. Targeted DamID replicate correlations.

Plot showing correlation across binding profiles of Dam:KDM5 TaDa replicates.

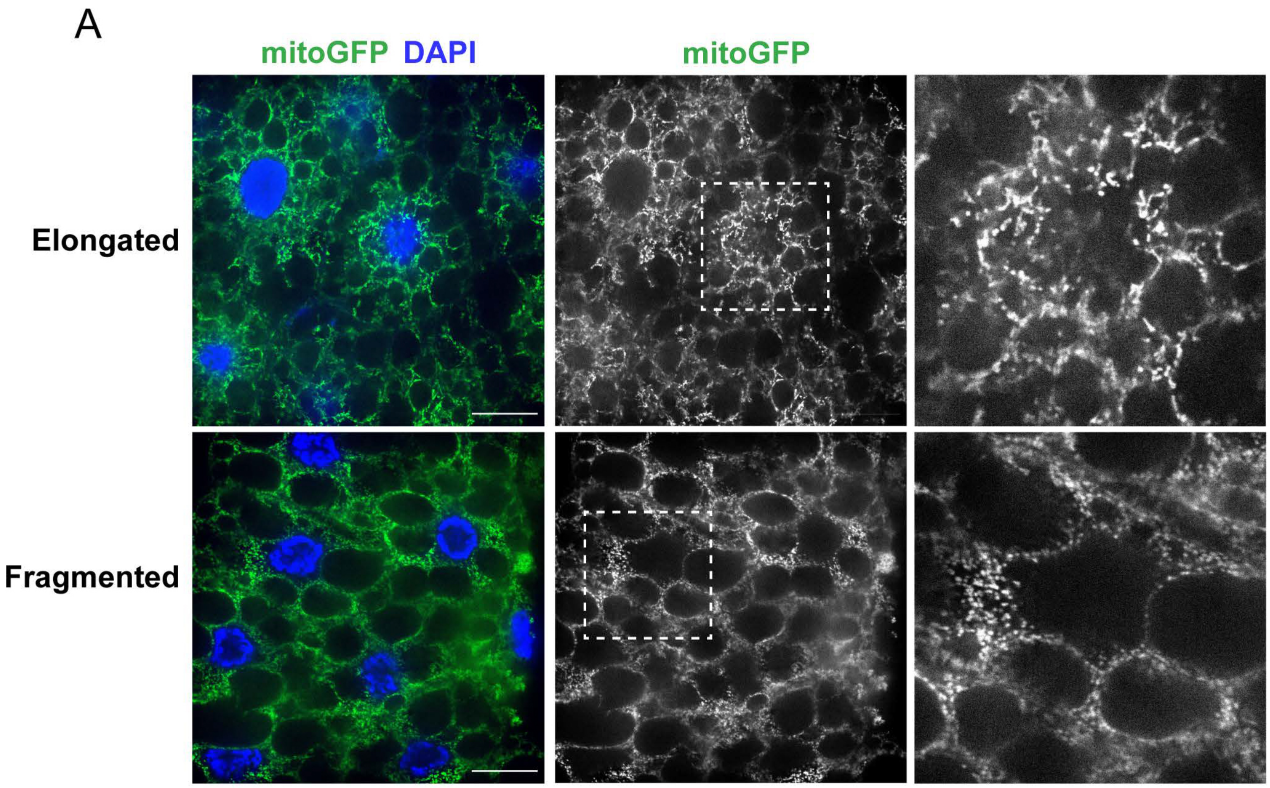

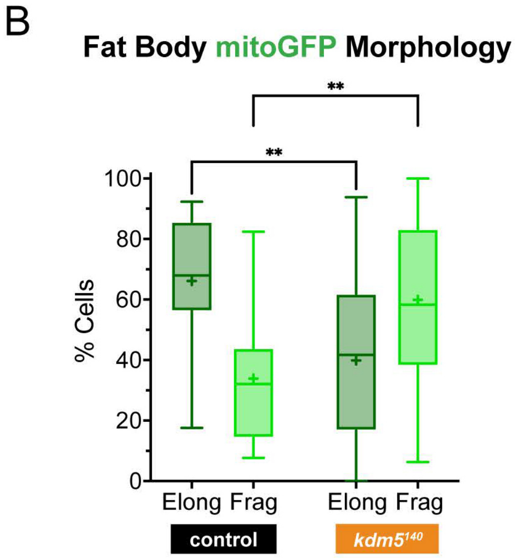

Fig. S3. KDM5 affects mitochondrial morphology in the fat body.

(A) Single Z slices of larval fat body cells expressing *CG>mitoGFP* with elongated and fragmented morphologies, respectfully. Zoomed images highlight representative single cells. Scale bars: 20 µm.

(B) Quantification of mitoGFP morphological classifications normalized to number of cells. Control genotype: *kdm5*^{140}/CyO-GFP. n= 14, 15, respectively. **p<0.01 (nonparametric unpaired t test). Error bars: mean + s.e.m.

Table S1. TaDa-identified KDM5 target genes.

Available for download at

https://journals.biologists.com/dev/article-lookup/doi/10.1242/dev.202024#supplementary-data

Table S2. RNA-seq from kdm5[140] ring glands.

Available for download at

https://journals.biologists.com/dev/article-lookup/doi/10.1242/dev.202024#supplementary-data

Table S3. full list of genes identified by GO DAVID Gene Ontology.

Available for download at

https://journals.biologists.com/dev/article-lookup/doi/10.1242/dev.202024#supplementary-data

Table S4. Key resources

Available for download at

https://journals.biologists.com/dev/article-lookup/doi/10.1242/dev.202024#supplementary-data

Table S5. Fly genotypes

Available for download at

https://journals.biologists.com/dev/article-lookup/doi/10.1242/dev.202024#supplementary-data
