# Supplementary material for: KDM5-mediated activation of genes required for mitochondrial biology is necessary for viability in Drosophila
Source: Development. 2023 Nov 6;150(21):dev202024. doi: 10.1242/dev.202024 (PMC10651110; doi:10.1242/dev.202024)
Supplement: Supplementary file 1 [file develop-150-202024_review_history.pdf]

KDM5-mediated activation of genes required for mitochondrial biology is necessary for viability in Drosophila

Michael F. Rogers, Owen J. Marshall and Julie Secombe
DOI: 10.1242/dev.202024

Editor: Swathi Arur

Review timeline

Original submission: 20 May 2023
Editorial decision: 19 June 2023
First revision received: 11 September 2023
Accepted: 29 September 2023

Original submission

First decision letter

MS ID#: DEVELOP/2023/202024

MS TITLE: KDM5-mediated activation of genes required for mitochondrial biology is necessary for viability in Drosophila

AUTHORS: Michael F Rogers, Owen J Marshall, and Julie Secombe

I have now received all the referees' reports on the above manuscript, and have reached a decision. The referees' comments are appended below, or you can access them online: please go to BenchPress and click on the 'Manuscripts with Decisions' queue in the Author Area.

As you will see, the referees continue to express excitement about the work. However, there are two key points that need to addressed before we can consider publishing the work. First, all three reviewers while excited about the findings suggest that a key experiment that needs to be conducted to test the role of KDM5 in the prothoracic gland specifically to regulate mitochondrial biology is a rescue experiment, wherein KDM5 is specifically driven in the prothoracic gland and rescues the phenotypes. Second, reviewer 3 provides suggestions on how some of the mitochondrial analysis can be better presented and analyses to add further rotor to the analysis. I agree with both these suggestions as being important to elevate the rigor in the analysis of an exciting study. I invite you to submit a revised version of the manuscript. Your revised paper will be re-reviewed by one or more of the original referees. Please send us a point-by-point response indicating your plans for addressing the referee’s comments, and we will look over this and provide further guidance.

Please attend to all of the reviewers' comments and ensure that you clearly highlight all changes made in the revised manuscript. Please avoid using 'Tracked changes' in Word files as these are lost in PDF conversion. I should be grateful if you would also provide a point-by-point response detailing how you have dealt with the points raised by the reviewers in the 'Response to Reviewers' box. If you do not agree with any of their criticisms or suggestions please explain clearly why this is so.

Reviewer 1

Advance summary and potential significance to field

© 2023. Published by The Company of Biologists under the terms of the Creative Commons Attribution License (https://creativecommons.org/licenses/by/4.0/).

In this manuscript, Rogers and others investigated functions of Lysine Demethylase 5 (KDM5) family proteins using Drosophila genetics. Drosophila encodes a single KDM5 gene, making it an ideal model system for investigating evolutionarily conserved functions of this chromatin modifier. Based on their own previous studies, the authors mainly focused on its function in the steroidogenic tissue called the prothoracic gland and identified its critical role in regulating mitochondrial physiology.

Overall, experiments are carefully designed and conducted, and results are mostly interpreted properly. The proposed model (Figure 6) is also discussed well to convince readers that the conclusion of this study has significant implications that go beyond Drosophila genetics and physiology. Having said that, I have several points that need the authors' attention as mentioned below.

Comments for the author

Major points:
1) Based on their own previous studies, the authors focused on the functions of KDM5 in the prothoracic gland pretty much throughout the manuscript. It therefore does not make sense that they used Ubi-Gal4 in Figure 2. I cannot think of any technical difficulties for performing the same set of experiments using spok-Gal4. The authors need to perform the experiments in Figure 2 using spok-Gal4 and examine the critical period when KDM5 is required in the prothoracic gland.
2) The authors examined mitochondrial morphology in the last set of experiments and showed that the shape of the organelle is significantly different between control and kdm5 mutants (Figure 5I). Although the result is exciting, it is currently not convincing enough, as the authors did not perform rescue experiments. The authors need to express transgenes in the prothoracic gland that can rescue lethality of kdm5 mutants (such as KDM5 itself, RasV12, and most importantly Ets97D) and test if they can also rescue mitochondrial morphological defects.

Minor points:
3) p. 11, lines 263-265, "In contrast… thus kdm5 expression is required as early as mid to late larval stages.": This data interpretation is incorrect. Based on their graph and diagram in Figure 2C, it is too late to rescue the lethality if kdm5 is expressed starting day7, which corresponds to early L2. So this results rather indicates that kdm5 expression is required by early L2. When they perform the same set of experiments using spok-Gal4, it is critical to interpret the data more accurately, as it affects the validity of experimental design in Figures 3-5.
4) Figure 5A: Set the maximum value of the x axis to 100%, not 175%.
5) Figure 5C: Control DAPI image is replaced with the MitoTracker image.
6) Figure 5H: The authors need to show bigger images of tubular, intermediate and fragmented mitochondria (panels 1-3). Currently they are too small to appreciate mitochondrial morphology.
7) Figure 6: The model is described as if histone methylation/demethylation is critical for KDM5 function in the prothoracic gland. But on p. 4, lines 96-98, the authors state that the enzymatic function of KDM5 is not required for its function in controlling lethality. This seeming discrepancy needs to be clarified.

Reviewer 2

Advance summary and potential significance to field

Rogers et al examine the role of the histone lysine demethylase KDM5 in Drosophila, focusing on prothoracic gland function. KDM5 genes are often mutated in developmental disorders and cancers, with examples of oncogene and tumor suppressor functions both being reported. The catalytic activity of KDM5 as a lysine demethylase has also been found to be dispensable for development in Drosophila. Hence, much remains to be learned about KDM5 function and the pleiotropic effects of its mutation. This laboratory has studied KDM5 extensively and they previously discovered that the lethal phenotype of whole animal KDM5 null mutants could be suppressed by activation of the MAPK pathway in a larval neurosecretory organ called the Prothoracic Gland (PG). Here, they continue their examination of the role of KDM5 in the PG, first by expanding the set of RTKs and effectors capable of suppressing KDM5 lethality when expressed in the PG at specific developmental stages. They then perform DNA binding profiling experiments using TaDa specifically in the PG (incredible since it consists of ~50 cells).

© 2023. Published by The Company of Biologists under the terms of the Creative Commons Attribution License (https://creativecommons.org/licenses/by/4.0/).

These profiling experiments are complemented by RNA-seq analysis of Ring glands which provides correlative evidence that KDM5 activates genes involved in mitochondrial function. Consistent with the GO signature, they find that mitochondria exhibit defective morphology in PG cells of KDM5 mutants. Overall the manuscript is exceptionally well-written, the authors present even-handed interpretation of their results, and the figures are clearly designed, making this manuscript accessible to a broad readership.

Comments for the author

Despite its multiple strengths, additional work is needed to meet the title's claim that activation of mitochondrial genes by KDM5 is necessary for viability. The first three figures only modestly advance understanding of KDM5 function beyond the lab's prior studies. For example, they already demonstrated MAPK pathway activation in the PG could rescue KDM5 viability. Even the TaDa data reveal very similar profiles as prior experiments from other tissues and stages (KDM5 binds promoters of ~50% of the genes in the genome), and hence do not provide deeper insight into KDM5 function. By contrast, the RNA-seq data begin to advance understanding further in Figure 4, but there is only one additional data figure included in the manuscript. Additional experiments are needed to support the claim that KDM5 mutants die because of mitochondrial defects in PG cells. The strongest evidence provided comes from partial rescue of viability by expression in PG cells of Ets97D, a transcription factor with homology to human TFs involved in nuclear control of mitochondrial function. However, it is unclear why Ets97D rescues because it is not downregulated in the RNA-seq data from KDM5 mutant ring glands. The paper's conclusions would be considerably strengthened if it could be shown that Ets97D expression rescued the observed mitochondrial defects in KDM5-mutant PG cells. Even better, RNA-seq of Ets97D-expressing Ring glands from KDM5 mutants would help to identify key target genes that rescue viability.
Alternatively, the authors could examine the impact of MAPK pathway activation on mitochondrial dysfunction in PG cells. For instance, does MAPK pathway activation improve mitochondrial defects in KDM5 mutants? Further connections between experimental genotypes that rescue viability and mitochondrial function are needed to strengthen the paper's main conclusions.

Minor comments:
- Are mitochondrial defects observed in other KDM5 mutant cells besides the PG, hence explaining the ability to suppress lethality with PG-specific transgene expression? The authors could examine other cell types with varied metabolic loads relative to PG cells.
- Are mitochondrial defects still observed in KDM5 catalytic-dead genotypes?
- Is insight into the role of KDM5 gained by examining PG-specific KDM5 target genes relative to other KDM5 profiling datasets? (Fig 3F, 969 PG-specific KDM5 bound genes)
- The DAPI channel in figure 5C is a duplication of the MitoTracker Red channel for Control.

<u>Reviewer 3</u>

Advance summary and potential significance to field

Here Rogers et al have demonstrated that KDM5 is necessary for the development and viability of prothoracic gland cells via positively regulating mitochondrial metabolism. KDM5 classical function is as a histone demethylase which repressed transcription by targeting H3K4me3. Emerging studies are highlighting that KDM5 has an important pro transcriptional role. This manuscript therefore also provides concrete evidence that essential activities of KDM5 includes activating gene expression, in particularly mitochondrial metabolism. genes.

Comments for the author

The manuscript presented by Rogers et al details the important role for KDM5 in Drosophila development.

Overall the study is rigorous, and makes novel contributions to how KDM5 and possibly mitochondrial metabolism are necessary for Drosophila development. My comments are largely focused on Figure 5, and related to the mitochondrial studies.

© 2023. Published by The Company of Biologists under the terms of the Creative Commons Attribution License (https://creativecommons.org/licenses/by/4.0/).

1.Does Ets97D restore the expression of any mitochondrial metabolism genes (seq or qRT-PCR)? This would provide evidence that the rescue of mito metabolism is responsible for the rescued phenotype.

2. In figure C, the mitotracker red is shown twice for control, once in place of DAPI.

3. The GFP and MTR show very interesting staining patterns. Is this consistent across multiple larvae? The reason this is important is that the values calculated in D-G might not represent the entire situation. In the dkm5140, there is little staining of mites in the center compared to the periphery. In some places the MTR is quite strong where there could be few mites. The ration of MTR/mitoGFP would indicate high activity which can be associated with stress. likewise in Fig H there's seems to be more intense staining around the periphery (are the images control or kdm5140)? it looks to me based on one image in C, that the homogeneity of mitos is disrupted. Further in the WT there is strong mito/MTR on specific edges. Is there biological insight into why these cells/regions might have higher activity and what the consequence could be if they are defective in the absence of KDM5?

4. As mentioned, given the heterogeneity of staining I donate think D-F is revealing. However you could calculate the MTR/MG ratio to give an overall ratio of activity/mito mass for each data point.

5. 5H. I am not convinced on the mito morphology based on the tiny figures. This is a key point as fissed or fragmented mitos are predicted to be less efficient at OXPHOS and fused (tubular) are more active.

6. There is heterogeneity in the MG staining as well. Does this correlate with a type of mitochondria and with anything mentioned above in pt 3?

7. Are there any sensors that can be used to detect ATP which would be the way to demonstrate cellular bioenergetics are deficient. normal ATP might suggest there is a compensatory glycolytic response.

Overall this is a great study and better characterization of the mitochondrial findings would improve the manuscript.

First revision

Author response to reviewers' comments

We thank the reviewers for enthusiasm for our work and their thoughtful comments and suggestions to improve our manuscript. Below we describe changes to the manuscript in response to each concern. We additionally highlighted important changes made in the manuscript file using orange text so that reviewers can easily see where alterations have been made.

Reviewer 1

Major Points

1) The authors need to perform the experiments in Figure 2 using spok-Gal4 [instead of Ubi-Gal4] and examine the critical period when KDM5 is required in the prothoracic gland.

We agree that spok-Gal4 would be the best driver for these experiments. We used the ubiquitous Ubi-Gal4 driver for technical reasons. Unfortunately, when we performed these experiments using the tubulin-Ga80ts and spok-Gal4-driven rescue of kdm5140, we did not observe rescued flies at 29°C. While we do not know the reason for this, we do note that the addition of the tubulin-Ga80ts transgene also decreases the efficiency of Ubi-Gal4-mediated rescue, despite similar levels of KDM5 protein being detected by western blot. Thus, since the spok-Gal4-driven rescue capacity is already limited (19.7%), we anticipate that the tubulin-Ga80ts transgene is

© 2023. Published by The Company of Biologists under the terms of the Creative Commons Attribution License (https://creativecommons.org/licenses/by/4.0/).

responsible for the lack of rescue within this experimental design. While imperfect, the Ubi>kdm5 experiments in Figure 2 confirmed that KDM5 has critical roles in larval stages, and this result supported studying KDM5 function in larvae. We are happy to provide the spok-Gal4 experimental data at the reviewer's request.

2) The authors need to express transgenes in the prothoracic gland that can rescue lethality of kdm5 mutants (such as KDM5 itself, RasV12, and most importantly Ets97D) and test if they can also rescue mitochondrial morphological defects.

We agree with the reviewer. To directly test our hypothesis that altered mitochondrial morphology in prothoracic gland cells is critical to the lethality caused by loss of kdm5, we now show that expressing Ets97D under the control of spok-Gal4 suppresses this phenotype. These data are shown in Figure 6B.

Minor Points:
3) p. 11, lines 263-265, "In contrast… thus kdm5 expression is required as early as mid to late larval stages.": This data interpretation is incorrect. Based on their graph and diagram in Figure 2C, it is too late to rescue the lethality if kdm5 is expressed starting day7, which corresponds to early L2. So this results rather indicates that kdm5 expression is required by early L2. It is critical to interpret these data more accurately, as it affects the validity of experimental design in Figures 3-5.

We understand the reviewer's concern with the interpretation of the data presented in Figure 2C. An important note towards this point is that the developmental stage schematics below the x-axes of Figures 2C and 2D represent average developmental windows of the variable kdm5140 developmental delay. Given the limitations of this experimental system, we are careful not to overinterpret the results of these data. We agree with the reviewer that lethality rescue is diminished after day 7, but we did not see any rescue at days in which kdm5140 animals were identified at L3 stage or later (beyond day 10). This indicated to us that kdm5140 animals would likely have critical defects present by mid to late larval stages, which we confirmed with wandering L3 larvae in Figures 4 and 6.

4) Figure 5A: Set the maximum value of the x axis to 100%, not 175%.

We agree that an axis maximum of 175% is inappropriate in isolation. However, since this experiment and quantification in Figure 5A was performed in the same manner as those in Figures 1D-H, we kept the graph axes consistent for direct comparison across all lethality experiments in the study.

5) Figure 5C: Control DAPI image is replaced with the MitoTracker image.
This has been corrected.

6) Figure 5H: The authors need to show bigger images of tubular, intermediate and fragmented mitochondria (panels 1-3). Currently they are too small to appreciate mitochondrial morphology.
This has been corrected.

7) Figure 6: The model is described as if histone methylation/demethylation is critical for KDM5 function in the prothoracic gland. But on p. 4, lines 96-98, the authors state that the enzymatic function of KDM5 is not required for its function in controlling lethality. This seeming discrepancy needs to be clarified.

We agree with the reviewer and have altered the model to more clearly reflect the fact that KDM5 plays critical roles in the prothoracic gland independently of its histone modifying enzymatic activity.

Reviewer 2

Major Point

The paper's conclusions would be considerably strengthened if it could be shown that Ets97D expression rescued the observed mitochondrial defects in KDM5-mutant PG cells.

We agree with the reviewer. To directly test our hypothesis that altered mitochondrial morphology in prothoracic gland cells is critical to the lethality caused by loss of kdm5, we now

© 2023. Published by The Company of Biologists under the terms of the Creative Commons Attribution License (https://creativecommons.org/licenses/by/4.0/).

show that expressing Ets97D under the control of spok-Gal4 suppresses this phenotype. These data are shown in Figure 6B.

Minor Points

Are mitochondrial defects observed in other KDM5 mutant cells besides the PG, hence explaining the ability to suppress lethality with PG-specific transgene expression?

We performed this experiment in larval fat body using CG-Gal4, and the data have been incorporated into the manuscript (Fig. S3).

Are mitochondrial defects still observed in KDM5 catalytic-dead genotypes?

We have not attempted to examine this, as, in contrast to kdm5140 animals, kdm5JmC* mutants (catalytic-dead) do not exhibit developmental delay nor lethality. Taken with our newly generated data showing that Ets97D expression suppresses both the mitochondrial defects and the lethality of kdm5140, we do not feel that examining kdm5JmjC* will provide critical new information for this study.

Is insight into the role of KDM5 gained by examining PG-specific KDM5 target genes relative to other KDM5 profiling datasets? (Fig 3F, 969 PG-specific KDM5 bound genes)
We felt that the scientifically rigorous approach to take with the analyses of our TaDa data was to compare these to TaDa and ChIP-seq datasets we have generated from other tissues. This revealed that KDM5 binds to some target gene across cell types, in addition to showing some tissue-specific promoter recruitment. The kdm5140 lethality could be a result of the dysregulation of PG specific target genes, universal targets, or a combination of the two.

The DAPI channel in figure 5C is a duplication of the MitoTracker Red channel for Control.
This has been corrected.

Reviewer 3

1) Does Ets97D restore the expression of any mitochondrial metabolism genes (seq or qRT-PCR)? This would provide evidence that the rescue of mito metabolism is responsible for the rescued phenotype.
While this request would be feasible for other tissues, ring glands are comprised of ~50 cells and so are very difficult and labor intensive to dissect. It took 3-4 dedicated months of dissections to obtain sufficient material for the ring gland samples from kdm5140 and control animals for the RNA-seq study, and a comparable amount of RNA would be required for qPCR. We would therefore argue that we have already provided sufficient data to support our model: Known target genes of Ets97D show reduced expression in kdm5140, and expression of Ets97D in the prothoracic gland suppresses the physical mitochondrial defects and the lethality of kdm5140.

2) In figure C, the mitotracker red is shown twice for control, once in place of DAPI.

This has been corrected.

3) The GFP and MTR show very interesting staining patterns. Is this consistent across multiple larvae? The reason this is important is that the values calculated in D-G might not represent the entire situation. In the kdm5140, there is little staining of mites in the center compared to the periphery. In some places the MTR is quite strong where there could be few mites. The ration of MTR/mitoGFP would indicate high activity which can be associated with stress. likewise in Fig H there's seems to be more intense staining around the periphery (are the images control or kdm5140)? it looks to me based on one image in C, that the homogeneity of mitochondria are disrupted. Further in the WT there is strong mito/MTR on specific edges. Is there biological insight into why these cells/regions might have higher activity and what the consequence could be if they are defective in the absence of KDM5?
We also noticed this. However, we are currently unsure whether the differences in staining intensity across the prothoracic gland are biologically meaningful or whether they are a technical artifact. Very little is known about how cells within the prothoracic gland behave individually and/or jointly to regulate ecdysone production and secretion. As can be seen from our mitoGFP staining (Figure 6A), individual cells seem to show relatively consistent mitochondrial morphology

© 2023. Published by The Company of Biologists under the terms of the Creative Commons Attribution License (https://creativecommons.org/licenses/by/4.0/).

that can be distinct from adjacent cell(s). A similar phenomenon is observed with respect to DNA replication, which is triggered in prothoracic gland cells asynchronously. As a result, we would expect different PG cells in larvae to be at different stages in the cell cycle and likely also have different metabolic responses to these mitochondrial defects/stress.

4. As mentioned, given the heterogeneity of staining I donate think D-F is revealing. However you could calculate the MTR/MG ratio to give an overall ratio of activity/mito mass for each data point.
We performed this calculation and have added this data to Figure 5H. Similar to the comparisons in Figures 5D-G, there does not appear to be any difference at a tissue level for MTR/MG ratio in kdm5140 prothoracic glands compared to wild type.

5. In Figure 5H, I am not convinced on the mito morphology based on the tiny figures. This is a key point as fissed or fragmented mitos are predicted to be less efficient at OXPHOS and fused (tubular) are more active.
This has been corrected.

6. Are there any sensors that can be used to detect ATP which would be the way to demonstrate cellular bioenergetics are deficient. normal ATP might suggest there is a compensatory glycolytic response.

While directly assessing ATP levels would add to our story, we were unable to find any currently available reporters that would facilitate this analysis of ATP on an individual cell level within the prothoracic gland.

Second decision letter

MS ID#: DEVELOP/2023/202024

MS TITLE: KDM5-mediated activation of genes required for mitochondrial biology is necessary for viability in Drosophila

AUTHORS: Michael F Rogers, Owen J Marshall, and Julie Secombe
ARTICLE TYPE: Research Article

I am happy to tell you that your manuscript has been accepted for publication in Development, pending our standard ethics checks.

Reviewer 1

Advance summary and potential significance to field

In this manuscript, Rogers and others investigated functions of Lysine Demethylase 5 (KDM5) family proteins using Drosophila genetics. Drosophila encodes a single KDM5 gene, making it an ideal model system for investigating evolutionarily conserved functions of this chromatin modifier. Based on their own previous studies, the authors mainly focused on its function in the steroidogenic tissue called the prothoracic gland and identified its critical role in regulating mitochondrial physiology.

Comments for the author

The authors have addressed my concerns for the previous version of the manuscript.

Reviewer 2

Advance summary and potential significance to field

© 2023. Published by The Company of Biologists under the terms of the Creative Commons Attribution License (https://creativecommons.org/licenses/by/4.0/).

The authors examine the role of KDM5 in the PG, first by expanding the set of RTKs and effectors capable of suppressing KDM5 lethality when expressed in the PG at specific developmental stages. They then perform DNA binding profiling experiments using TaDa specifically in the PG (incredible since it consists of ~50 cells).

These profiling experiments are complemented by RNA-seq analysis of Ring glands which provides correlative evidence that KDM5 activates genes involved in mitochondrial function. Consistent with the GO signature, they find that mitochondria exhibit defective morphology in PG cells of KDM5 mutants. Lastly Ets97D expression rescued the observed mitochondrial defects in KDM5-mutant PG cells.

Comments for the author

the authors have satisfactorily addressed my comments.

Reviewer 3

Advance summary and potential significance to field

This manuscript advances out understanding of the regulation of mitochondrial biology by KDM5. This is significant because people with a pathological variants of KDM5C, a mammalian orthologue suffer from a variety of physiological alterations with varying intensity. While there are 4 orthologues in humans, KDM5C is emerging as a key regulator of mitochondrial metabolism. The positive regulation of KDM5/C of these genes remains a bit of a mystery since the canonical activity of KDM5/C is to repress gene expression. Research into this mechanism, such as this paper, will ultimately lead to an understanding of how a "transcriptional repressor" promotes gene expression in certain contexts. This will ultimately change the lives of people with KDM5C variants.

Comments for the author

The revised manuscript has been improved and the reviewers comments have been adequately addressed.

© 2023. Published by The Company of Biologists under the terms of the Creative Commons Attribution License (https://creativecommons.org/licenses/by/4.0/).
